# Heart Rate Variability in Children and Adolescents with Cerebral Palsy—A Systematic Literature Review

**DOI:** 10.3390/jcm9041141

**Published:** 2020-04-16

**Authors:** Jakub S. Gąsior, Antonio Roberto Zamunér, Luiz Eduardo Virgilio Silva, Craig A. Williams, Rafał Baranowski, Jerzy Sacha, Paulina Machura, Wacław Kochman, Bożena Werner

**Affiliations:** 1 Faculty of Medical Sciences and Health Sciences, Kazimierz Pulaski University of Technology and Humanities, 26-600 Radom, Poland; 2 Departamento de Kinesiología, Universidad Católica del Maule, 3480112 Talca, Maule, Chile; beto.zam@gmail.com; 3 Department of Internal Medicine, Ribeirão Preto Medical School, University of São Paulo, Ribeirão Preto, São Paulo 14049-900, Brazil; luizeduardovs@gmail.com; 4 Children’s Health and Exercise Research Centre, Sport and Health Sciences, College of Life and Environmental Sciences, University of Exeter, St Luke’s Campus, Exeter EX1 2LU, UK; c.a.williams@exeter.ac.uk; 5 Department of Heart Rhythm Disorders, National Institute of Cardiology, 04-628 Warsaw, Poland; rb@ikard.pl; 6 Faculty of Physical Education and Physiotherapy, Opole University of Technology, 45-758 Opole, Poland; sacha@op.pl; 7 Department of Cardiology, University Hospital in Opole, University of Opole, 45-401 Opole, Poland; 8 Department of Gynaecological Endocrinology, Medical University of Warsaw, 00-950 Warsaw, Poland; paulinaolesinska28@gmail.com; 9 Clinical Department of Cardiology at Bielanski Hospital, National Institute of Cardiology, 01-809 Warsaw, Poland; w.kochman@icloud.com; 10 Department of Pediatric Cardiology and General Pediatrics, Medical University of Warsaw, 02-091 Warsaw, Poland; bozena.werner@wum.edu.pl

**Keywords:** cerebral palsy, heart rate variability, cardiac autonomic dysfunction

## Abstract

Cardiac autonomic dysfunction has been reported in patients with cerebral palsy (CP). The aim of this study was to assess the existing literature on heart rate variability (HRV) in pediatric patients with CP and a special attention was paid to the compliance of the studies with the current HRV assessment and interpretation guidelines. A systematic review was performed in PubMed, Web of Science, and Cumulative Index to Nursing and Allied Health Literature (CINAHL) databases searched for English language publications from 1996 to 2019 using Medical Subject Headings (MeSH) terms “heart rate variability” and “cerebral palsy” in conjunction with additional inclusion criteria: studies limited to humans in the age range of 0–18 years and empirical investigations. Out of 47 studies, 12 were included in the review. Pediatric patients with CP presented a significantly higher resting heart rate and reduced HRV, different autonomic responses to movement stimuli compared to children with normal development, but also reduced HRV parameters in the children dependent on adult assistance for mobility compared to those generally independent. None of the included studies contained the necessary details concerning RR intervals acquisition and HRV measurements as recommended by the guidelines. Authors of HRV studies should follow the methodological guidelines and recommendations on HRV measurement, because such an approach may allow a direct comparison of their results.

## 1. Introduction

Cerebral palsy (CP) is a neurodevelopmental condition starting in early childhood and lasting for the whole life. In patients with CP, motor disorders attributed to non-progressive anomalies in the developing fetal or infant brain are often accompanied by a range of additional disturbances, e.g., epilepsy, sensory, perceptive, cognitive, communicational, and behavioral problems [1]. Recently, numerous studies have been performed to identify alterations in the autonomic nervous system (ANS) activity, which suggest that children and adolescents with CP present sympathovagal imbalance compared to peers with normal development [2,3,4,5,6,7,8,9,10,11,12,13,14]. Several possible causes are postulated to explain the cardiac ANS abnormalities in these patients. Firstly, the brain injury affects parts of the ANS and hence impairs cardiovascular functioning, e.g., heart rate (HR) regulation [15,16,17,18], which in turn may lead to cardiovascular complications [19,20,21,22]. Secondly, mobility limitations, lack of physical activity, and, in general, a sedentary lifestyle observed in this population undermine the ANS and potentiate the risk of cardiometabolic diseases [23,24]. Finally, the overall regulation of the cardiovascular system is disturbed, which results in elevated blood pressure and HR.

Heart rate variability (HRV) analysis is a noninvasive measure of the cardiac ANS modulation, which is widely used to reflect heart-brain interactions [25,26]. Since 1996, when the Task Force of the European Society of Cardiology and the North American Society of Pacing and Electrophysiology published standards of measurement, physiological interpretation, and clinical use of HRV [25], a large number of research and methodological papers in the area of HRV have been published [27,28,29,30,31,32,33,34,35,36,37,38,39,40,41,42,43,44,45,46]. In general terms, reduced HRV indicates ANS imbalance and a poor prognosis in patients with a variety of clinical conditions [47,48,49,50]. Indeed, reduced HRV is often seen in diabetic children with autonomic neuropathy [51,52], and in those with a clustering of metabolic risk factors [53]. In 2014, a review assessing the function of the autonomic HR regulation among children with CP was published [10]. Results of the studies included to the review (articles published before 2013) performed by Amichai and Katz-Leurer [10], but also findings of the articles that were not analyzed by these authors or were published later, showed that children with CP, in comparison to normally developing peers, present a decreased overall HRV profile, an increased average HR, and altered autonomic responses to selected movement maneuvers or physical exercise [4,6,8,9,10,11,12,14,54,55,56]. However, results of the mentioned HRV analyses in children with CP are not fully concordant, which may be partially caused by different methodologies and a lack of compliance with the recommendations of the Task Force [25]. Another important point to be considered is that there are several limitations regarding the overall assessment and analysis of HRV that can lead to heterogenous results [34]. Thus, the assessment of whether the studies of HRV in children with CP followed the recommendations for obtaining RR intervals and performing HRV analysis is critical for summarizing the current knowledge and for planning future research. Characterization of the cardiac autonomic control in children with CP is paramount for better understanding of neurological disturbances and their development in this condition. Furthermore, by summarizing the effects of interventional studies on HRV, it will be possible to define the role of HRV analysis in monitoring the autonomic activity in subjects with CP undergoing various modes of rehabilitation and treatment. In addition, to the best of our knowledge, no review has addressed compliance of the previously published studies with the proposed methodological recommendations for the analysis and interpretation of HRV in this population.

Therefore, the aim of this study was to review the existing literature on HRV in pediatric patients with CP with a particular attention to verifying compliance of the included studies with the current guidelines for HRV analysis.

## 2. Literature Search

A systematic search of the literature according to the evidenced-based and consensus-based Preferred Reporting Items for Systematic Reviews and Meta-Analyses (PRISMA) statement [57,58] was carried out. Three digital databases (PubMed, Web of Science and Cumulative Index to Nursing and Allied Health Literature (CINAHL) were searched for the studies reporting measurements of HRV in patients with CP. The databases were explored using mesh terms “heart rate variability” and “cerebral palsy” in conjunction with the following limitations: (i) studies limited to humans; (ii) studies limited to participants in the age range of 0–18 years; (iii) English language of the publication; (iv) publication date from March 01, 1996 (date of publication of the standards for HRV analyses [25]), to July 31, 2019; (v) empirical investigations, i.e., studies involving active data collection. Meta-analyses, expert opinions, reviews, and single case reports were excluded.

The StArt (State of the Art through Systematic Review) software (developed by the Federal University of São Carlos) was used to perform the systematic review.

The selected studies were independently analyzed by two researchers (J.S.G. and A.R.Z.), to minimize the bias. The disagreements between them were resolved through discussion with other co-authors. Titles and abstracts of all the initially identified articles were screened based on the defined inclusion criteria—in doubtful cases, full texts of the articles were analyzed. To broaden the research, references of the eligible articles were also searched for further relevant studies not identified in the database. Additionally, when it was impossible to download the full text of a relevant study, an e-mail with the request for the article was sent to the corresponding author.

According to the PICOS approach (each letter of the acronym PICOS refers to: the patient population or the disease being addressed (P), the interventions or exposure (I), the comparator group (C), the outcome or endpoint (O), and the study design chosen (S)) [57], the following information was extracted from each study: (1) first author and year of publication; (2) characteristics of participants (experimental group—patients with CP; control group—children with normal development); (3) all conditions concerning RR intervals acquisition; (4) method of HRV analysis; (5) results of HRV analysis, and (6) main results and conclusions.

The following quality aspects of HRV studies in pediatric patients with CP referring to the guidelines for HRV analysis were analyzed and reviewed:

- Study sample (the number of patients in the experimental group and of participants in the control group).
- Data acquisition and processing, where the following points are analyzed:
  − Device, software, duration of recordings, and sampling frequency;
  − Recording conditions: time of the day, room (lights/voices/temperature), activities before recordings (sleep routine, physical activities, meals, drinks, using the toilet), and heart rate stabilization;
  − Respiratory rate during recordings and breathing control;
- HRV analysis, where the following points are analyzed:
  − Software, artifact correction, time series length (time/beats), information about data normality;
  − Frequency domain and nonlinear HRV parameters;
- HRV correction for HR.

Detailed analysis and explanation of the relevance of each aspect is presented in the Discussion.

## 3. Results

**Figure 1:**
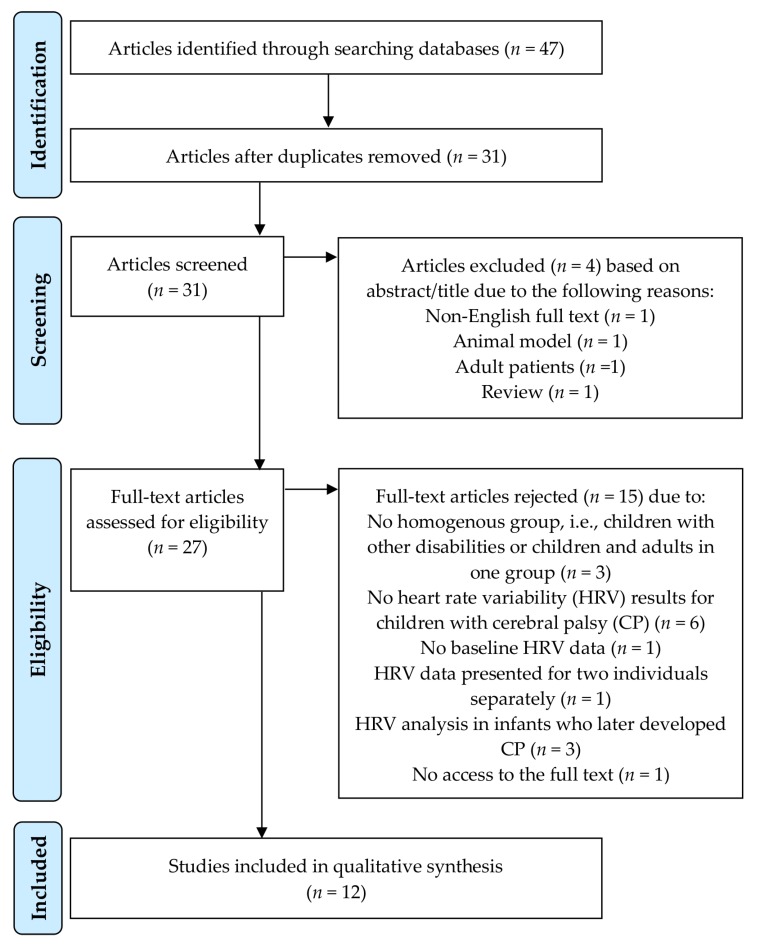
Preferred Reporting Items for Systematic Reviews and Meta-Analyses (PRISMA) flow diagram for the search process.

Data for points (1), (2), (3), and (4) are summarized in Table 1 and Table 2, respectively. Detailed results of the HRV analysis (5) reported in the included studies are presented in Table S1 (time-domain parameters; Supplementary Materials) and Table S2 (frequency-domain parameters; Supplementary Materials). Main results and conclusions (6) of the analyzed studies are presented in Table 3. The search procedure, i.e., the number of studies meeting the pre-defined inclusion criteria and the number of excluded and accepted studies is illustrated in Figure 1.

### 3.1. Selection of the Studies

The search of the databases yielded a total number of 47 articles (Figure 1). After removing duplicates, 31 articles remained—of these, 4 studies were discarded because they did not meet the established criteria. From the 27 remaining studies, after full text analysis and data extraction, 15 articles were excluded with specified reasons (see the flow diagram in Figure 1). A total of 12 studies were therefore included in the systematic review. No additional study was identified by checking the references within these 12 studies.

### 3.2. Information Provided by the Selected Studies

#### 3.2.1. Participants/Demographic Data

The included studies involved 397 participants with CP and were conducted in the period from 2002 to 2019. Details concerning age, sex, Gross Motor Function Classification System (GMFCS) level, and type of paralysis for patients with CP are presented in Table 1. Data describing the control group were available in 8 studies. In one study, patients with CP were considered the control group for children with acute brain injury (Table 1).

#### 3.2.2. RR Interval Recordings

Details concerning RR intervals acquisition are given in Table 1. Long-time recordings, i.e., 24-h ECGs, were performed in 1 study. Short-time RR interval recordings were used in 7 studies and the duration of recordings ranged from 3 to 15 min—in 4 studies, the length of RR interval recordings was not provided or there was lack of precise description. In 5 studies, the Polar Advanced Heart Rate Monitor was used. Sampling frequency varied within the range from 250 to 1000 Hz. The authors of 8 studies provided information about the software that was used to acquire the RR intervals. Comprehensive information about both sampling frequency and recording duration was provided in 3 studies. None of the studies employing short-term recordings provided comprehensive information and description on the time of data acquisition, conditions in the room where the study took place, the period preceding the recordings (i.e., the time needed for HR stabilization), and conditions before recordings (i.e., sleep routine, physical training, meals, drinks, using the toilet). In 3 studies, the breathing rate was controlled. In 7 studies, short-term recordings were performed in the supine position—in 6 of these studies, data were also recorded in other positions.

#### 3.2.3. HRV Measurement

Table 2 shows information about HRV measurements. One study provided details on the software used for calculation of HRV parameters, artifact correction and the length of the time series used for the HRV analysis. HRV parameters were calculated based on the time series length equal to 5 min (short-term recordings) or 18 h (long-term recordings). Two studies presented results of the time- and frequency-domain analyses and the nonlinear dynamics analysis. Three studies showed data concerning only frequency-domain parameters and 2 studies presented results of only time-domain parameters. In 3 articles, the authors specified how data distribution was checked.

#### 3.2.4. HRV Results

Detailed raw results reported in the included studies concerning HR, time-domain, frequency-domain, and nonlinear HRV parameters are presented in Tables S1 and S2, respectively. In Table 3, the main results and conclusions taken from these studies are presented.

## 4. Discussion

The present study had two parallel aims: to review the existing findings of HRV in pediatric patients with CP and to verify the compliance of these studies with the current guidelines for measurement, physiological interpretation, and clinical use of HRV.

Our main results revealed that children with CP present significantly higher resting HR and lower HRV and lower cardiac autonomic system adaptation to exercise and activity compared to typically developed children, but also decreased values of selected HRV parameters in those dependent on adult assistance for mobility compared to those who are generally independent. Physical exercises improve cardiac autonomic regulation in pediatric patients with CP by reducing HR and breathing rate and increasing values of selected HRV parameters. However, most of the studies reviewed did not follow the current guidelines on RR recording and HRV analysis or provided limited information concerning methodological aspects, thus limiting their reliability and replication.

HRV analysis provides an insight into the autonomic modulations of cardiac periodicity and consequently can be used to examine autonomic responsiveness [46]. However, after more than 30 years from the first studies on HRV, there is still resistance to using HRV as a tool for medical decision-making in the clinical practice. One of the reasons for this observation is the limitations/pitfalls of HRV analysis, which may lead non-experts in the field to misinterpret its results. Clarification of the limitations, drawbacks, and strengths of HRV is required to contribute to overcoming these methodological difficulties in future studies.

### 4.1. HRV Changes in Children with CP

Patients with CP have an increased risk of death, mainly due to the circulatory and respiratory systems diseases [59,60]. Therefore, it is important to examine cardiac, cardiorespiratory, and cardiac autonomic function in patients with CP to better understand the disease and to establish new therapeutic goals for this condition.

Many neurological disorders associated with brain damage, e.g., Parkinsonian syndrome, multiple sclerosis, and Guillain-Barré syndrome, are associated with decreased HRV values [61]. The results and conclusions of the studies included in this review also highlighted that patients with CP are characterized by impaired cardiac ANS (see Table 3). In 2014, Amichai and Katz-Leurer published a review based on the articles published between 1990 and 2013 assessing the function of the autonomic cardiac regulation among children with CP [10]. However, since that period, eight new articles in this area have been published [8,9,11,12,13,14,54,56]. Amichai and Katz-Leurer concluded that there is a trend that children with CP suffer from impaired cardiac autonomic system regulation [10]. In the supine position, patients with CP presented significantly higher resting HR and reduced HRV, but also different responses to stimuli, such as selected movement maneuvers (posture change, head-up tilt, or standing), compared to typically developed children. Results of the articles published between 2013 and 2019 confirmed this trend, but also provided new findings (see Table 3). Authors of these studies suggested that children with CP present lower cardiac autonomic system adaptation to exercise and activity [8] and lower spirometry [12] compared to typically developed children; non-optimal HR reduction after a submaximal treadmill test indicating low reactivation of the parasympathetic system [11]; decreased values of selected time-domain HRV parameters in those dependent on adult assistance for mobility compared to those who are generally independent [14]. Israeli-Mendlovic et al. [9] suggested and Cohen-Holzer et al. [13] and Amichai et al. [12] showed that some forms of physical exercises and training improve cardiac autonomic regulation in pediatric patients with CP by reducing HR and breathing rate and increasing values of selected HRV parameters.

Impaired cardiac autonomic function in children with CP might be due to the damage of the developing brain or to the sedentary lifestyle, or both synergistically interacting [10,12]. Recently, the association between the anterior cingulate cortex (ACC), a bilateral cortical structure in the medial wall of the brain [62], and HRV has been shown [63,64]. Authors reported that increases in HRV were accompanied by increases in functional connectivity between amygdala and dorsal ACC [65], the existence of positive associations between ACC cortical thickness and differences in vagally-mediated HRV [63] and between cerebral blood flow in the ACC and the high frequency component of HRV [64]. Importantly, it was shown that in children with CP, structural connectivity to the ACC was reduced [62,66].

In apparently healthy children, longer time spent in the sedentary lifestyle, lower physical activity and cardiorespiratory fitness levels were associated with poorer cardiac ANS function [67]. Many children with CP presented lower physical activity and cardiorespiratory fitness levels than their typically developed peers [68,69]. Moreover, in children with CP, sedentary lifestyle begins very early, i.e., in the childhood years [70]. Thus, the impaired autonomic modulation of cardiac oscillations in children with CP may be related to both the alterations attributed to disturbances occurring in the specified areas of developing brain, and prolonged sedentary behaviors.

### 4.2. Compliance of the Studies to Recommendations and Guidelines

Quintana and Heathers suggested that uncontrolled variables within experimental environments may significantly influence HRV results [34]. Careful consideration of such crucial contextual, environmental, physiological, and methodological factors is required to build more precise research protocols and ensure researchers obtain more accurate and reproducible results. Examples of such factors are the time of the day to record the data (in the case of short-time recordings); subject-characteristic variables such as age, sex, HR, breathing rate, health and physical activity status, control for medication, food and water consumption, voiding of the bladder; position of the body during short-time recordings; the quality of recorded signals (recording period length, detection or recording method, sampling frequency, breathing pacing—paced or free breathing); as well as the tools used to analyze HRV values (i.e., how HRV metrics were calculated—software, removal of artifacts, frequency band cutoffs, power spectral analysis method, etc.) [33,34,37,40,61,71].

#### 4.2.1. Study Sample

The studies that met the inclusion criteria involved 397 pediatric participants with CP (number of children with CP in experimental groups ranged from 10 to 110 participants). In seven studies, results for the control group (mostly typically developed children) were also presented. The number of typically developed children was the same or smaller compared to the experimental group, i.e., from 12 to 35 participants. It should be emphasized that in many studies, participants with CP were divided into sub-groups according to the GMFCS, which decreased the total number of participants in selected groups [8,9,14].

The distribution analysis of 297 HRV effect sizes from between-group/case-control studies performed by Quintana [72] highlighted that the Cohen’s guidelines [73] may underestimate the magnitude of small and large effect sizes. Therefore, it is suggested that HRV studies were generally underpowered. In case-control HRV studies, for vagally-mediated HRV measures, in order to achieve 80% of statistical power, samples of 233, 61, and 21 participants are required to detect small (effect size of 0.25), medium (effect size of 0.5), and large (effect size of 0.9) effect sizes, respectively (significance criterion of alpha = 0.05) [40,72]. Based on this statistical consideration, large effect size is observed in all the studies included in this review. Future investigations should include a larger pediatric population of patients with CP to confirm previous results based on smaller samples.

#### 4.2.2. Data Acquisition and Processing

- Device, software, duration of recordings, and sampling frequency

Authors of eight studies analyzed in this review specified the name of the software that was used to record the raw signals and obtain the RR intervals: in two articles, the electrocardiography was collected by authors using an electrocardiogram (ECG) device; whilst in seven studies, a heart rate monitor was used; in the other three articles, there was no information about the name of the device and the software used or the authors stated that they used their own software. In HRV studies, details on the raw signal acquisition device and the software used for RR interval generation and analysis should be provided. If the information on the acquisition device or the software used to calculate HRV is not available, a more precise methodological specification should be ensured to allow reproducibility [35].

RR interval data are generated via ECG (more traditionally) or, more recently, by photoplethysmography (PPG) [35,39]. Although ECG and PPG presented discrepancies of about 6% for most HRV measurements [74], selected PPG devices showed satisfactory agreement with ECG in some, but not all populations [75,76,77]. In terms of identification and correction of artifacts and ectopic beats, and also of the correct identification of cardiac events, e.g., cardiac dysrhythmia, ECG is more accurate than PPG [35,40]. The use of traditional ECG to obtain RR intervals in patients with CP in, e.g., a pre-post intervention study with stable conditions is relatively feasible. However, since ECG recording during rehabilitation/exercise activities in this population may be problematic, heart rate monitors could be used more freely during such activities. It was recently confirmed that in a healthy population, popular heart rate monitors are valid methods to detect RR intervals [78] and to record HRV [79]. In future studies, it should be verified whether heart rate monitors can produce ECG comparable to conventional devices, and reliable RR intervals (and, consequently, HRV measurements, for details see [80]) in children with CP in different conditions.

The information about the duration of recordings and the RR series length used to calculate HRV parameters should be provided separately and distinctly, because they do not represent the same information. Twenty-four-hour ECG recording was performed in one study included in this review. The author of the study clearly stated that the NN intervals over a period of at least 18 h were analyzed. The duration of recordings in the other 11 studies ranged between 3 and 15 min. In one study, the period of time of the RR interval series and the period of time used to calculate HRV parameters was the same (288 s); in other studies, where short-term recordings were analyzed, the 5 min time interval was used to calculate HRV parameters. However, none of the analyzed studies reported the number of points of RR series. Since the number of points of RR series is a function not only of time, but also of HR, and considering that robustness of some HRV analysis methods depends on the RR series length (such as nonlinear approaches), this information should be reported.

Sampling frequency was provided only in four of the 12 analyzed studies (from 250 to 1000 Hz). Sampling frequency is an important issue, and even if HR monitoring systems are used, it should be provided, since it has a direct effect on the resolution of RR intervals, thus limiting assessment of the actual HRV. Lower signal sampling rate (i.e., < 250 Hz) decreases the validity of HRV parameters, mostly of frequency-domain and nonlinear indices [25,37,39,40,81]. It has been suggested that sampling rates lower than 250 Hz, but not lower than 100 Hz, may be acceptable if appropriate interpolation algorithms are used [39]. The sampling rate between 250 and 500 Hz was recommended by the Task Force experts; however, other researchers suggest a sampling rate between 500 and 1000 Hz [40].

- Recording conditions: time of the day, room (lights/voices/temperature), activities before recordings (sleep routine, physical activities, meals, drinks, using the toilet), and heart rate stabilization

A number of environmental/external factors should be controlled in HRV experiments. It has been extensively addressed by some authors, e.g., by Quintana and Heathers (2014) [34], Heathers (2014) [33], Fatisson et al. (2016) [61], and Laborde et al., (2017) [40]. Seven of the analyzed studies in this review provided limited information on the conditions during signal acquisition, mostly concerning short-term recordings, as it is difficult to ensure all details on 24-h recordings. There was no study (in case of short-term recordings) with a full description of the time of the day when recording was performed, conditions concerning the room where the study took place and participant activities before recordings. Those conditions should be provided and, if possible, standardized for all participants. Laborde et al., (2017) [40] provided a useful demographic questionnaire that helps to collect and control most of the confounding variables influencing HRV ([40] Supplementary Materials (Data Sheet 1)). 

Only in four studies was the exact time planned for rest/HR stabilization before starting data recording provided, and it ranged from 5 to 30 min. HR is generally unstable and changes over time and after postural changes [34,82]. Therefore, a minimal steady HR signal is required for the analysis of short-term HRV and it is usually obtained by an appropriate time allowed for volunteers to acclimatize to the recording environment process. This process usually consists of a resting period of 5–10 min before the experiment begins [35,39,40]. However, some authors suggested that in some populations, even shorter acclimatization time periods may be implemented [83]. During baseline recordings, participants should be instructed to remain still without speaking [40]. Not speaking during the recording is particularly important, because it eliminates the respiratory component of HRV (respiratory sinus arrhythmia). Only in one study the children were asked not to talk or to move during data registration. In three studies, participants were instructed how to breathe during data collection.

- Respiratory rate during recordings and breathing control

HRV (mostly frequency-domain parameters) is affected by respiratory depth and frequency [34,84,85]. The low and high frequency bands (LF and HF, respectively) of HRV are affected by breathing when the patient breathes in a rate of ~3 to 9 and 9 to 24 breaths per minute, respectively [37,40,43]. The frequency band for HF was standardized to 0.15–0.40 Hz; however, for pediatric subjects, who breathe faster than adults [86], the range should be appropriately adjusted [35,37,40]. As the spectral powers at LF and HF bands are commonly attributed to sympathetic and vagal influences on the HR, the investigator should be aware that changes in the respiratory frequency may led to the invalidation of spectral indices. For instance, when any subject breathes very slowly, in the range of the LF band (~3 to 9 breaths per minute), the classical interpretation of the HF band as the vagal influence on the HR is flawed.

Breathing rate was measured in three studies concerning HRV in children with CP. The children were instructed to breathe with a metronome at 15 breaths/min [2] or maintained spontaneous breathing, presenting 10 to 20 breaths per minute [5]. In a recent study, Amichai et al. assessed the impact of the breathing rate on the cardiac autonomic dynamics in children with CP and in typically developed children [12]. The pacing procedure assumed slow and deep inhalation through the nose to a count of four, followed by a slow and complete exhalation to a count of six. The procedure was supported by watching the breathing waves on the screen and attempting to draw prolonged waves in each breathing cycle [12]. In both conditions, i.e., during rest and paced breathing, there were no significant differences in the respiratory rate between children with CP and typically developed children [12]. A significant reduction in the breathing rate during paced breathing noted within each group was accompanied by a nominal decrease in HR and a significant increase in the standard deviation of NN intervals (SDNN) and the root mean square of successive difference intervals (RMSSD) [12].

It is important to question whether changes in HRV parameters were associated primarily with changes in respiratory rate or changes in HR [87]. Even though HRV alterations resulting from changes of the respiration pattern are expected, knowing the respiratory rate is needed to establish whether the changes in HRV values are primarily due to changes in respiratory frequency or not [40], especially in populations that present different breathing frequencies or when an experimental task modifies the respiratory pattern [34,35]. 

It still remains that there is no optimal solution on how to record and control respiratory rate in HRV studies [34]. Quintana and Heathers proposed to measure the subject’s natural/normal respiration rate, and use the obtained frequency for respiratory pacing in the resting state registration [34]. Another solution, especially helpful in populations composed of children, is to perform acclimatization to the recording environment, which is intended to stabilize the respiration rate. This procedure consists of placing the individual in the position of the recording for a minimum period of time in order to stabilize both HR and respiration. Then, the respiratory rate can be derived from the ECG using proper algorithms [88] or be simultaneously recorded with the ECG using a respiratory belt. In another scenario, where an HR monitor is used to record RR intervals, authors may monitor respiratory rate using a camera (e.g., [89]). Subsequently, exclusion of participants with a respiratory rate out of the range established by the statistical analysis or changing the HRV frequency bands is warranted. Importantly, the choice of spontaneous or paced breathing during the recordings will have different consequences for HRV analysis and should be taken into account by researchers [90,91,92,93,94]. 

#### 4.2.3. HRV Analysis

- Software, artifact correction, time series length (time/beats), information about data normality

Only one of the twelve studies included in the presented review provided all the details on the software used to calculate HRV parameters, the artifact correction method, and the time series length used to calculate HRV indices. As mentioned above, details on how HRV parameters were calculated, as well as the software used for RR interval analysis should be routinely provided. We propose that a detailed description of the methods should be presented even when the software used in the analysis is reported.

Artifacts may significantly influence values of HRV parameters [27,37,95,96]. Before HRV parameters can be calculated, preprocessing of the raw data is necessary and should also be described. Data reduction/replication and cleaning procedure should be explained and justified. All abnormal beats (i.e., not generated by sinus node depolarization) and artifacts (e.g., missed or spurious beats) should be identified and properly processed [27,33,35,39,45,97,98].

The length of the RR intervals or the HR series is methodologically relevant, especially in the protocols that are dependent on the number of samples [39]. Different groups of authors have proposed various durations needed to obtain reliable time- and frequency-domain parameters [37,39], as well as nonlinear approaches [99,100,101]. However, it is commonly recommended, according to the Task Force paper [25], to calculate linear HRV short-term parameters from a 5 min time interval to ensure adequate comparison between studies [40]. For nonlinear methods, the choice of the series length depends on the method under consideration. A reasonable general approach is to follow the corresponding original paper recommendations to allow comparisons between studies.

Another point to be considered regards the statistical analysis. Out of the articles included in the present review, only three stated that data distribution was checked. In many HRV studies, most parameters presented a non-normal distribution. When this is the case, to meet statistical requirements, non-parametric tests should be used, or a log transformation procedure be performed, and log transformed data should be presented and evaluated [40,102].

- Frequency domain and nonlinear HRV parameters

Two frequency domain parameters are widely used in HRV studies, i.e., LF and HF powers (and, consequently, the LF/HF ratio), with commonly used bands 0.04–0.15 Hz and 0.15–0.40 Hz, respectively [25,39,45]. Such bands were used in all (with one exception, and one study where the authors did not specify frequency bands) the studies concerning HRV in children and adolescents with CP included in the presented review. However, for the pediatric participants who breathe faster than 24 breaths per minute, the range for HF should be adequately set by increasing the upper limit of the HF band [35,37,40]. Quintana et al. recommended for these population to set the HF band width at 0.24–1.04 Hz at rest [35].

Fast Fourier transform and autoregressive spectral analysis are the most commonly used methods for frequency-domain HRV analysis [45]. However, results obtained by these analyses could not be considered interchangeable and compared to each other, at least in healthy subjects at rest [103]. A full description of which frequency-domain method was used with additional methodological assumptions, such as the windowing method, window length, and overlap, should be given [35]. Authors of five studies specified the frequency analysis method used to obtain frequency-domain parameters and only one author provided more details on sampling and windowing.

Frequency-domain HRV parameters can be expressed in absolute (ms^2^) or relative, normalized (nu), units [25,37]. It is recommended to present values in both units [40], as there are some limitations when using only one of them [33]. In general, the absolute power of the HF band can be considered a robust index of cardiac vagal modulation, but the absolute power of the LF band is not a pure index of cardiac sympathetic modulation. Some authors showed, using autonomic maneuvers, that the normalized power in the LF band represents a more robust index of cardiac sympathetic control [104]. In two studies, authors presented data in both units; in two studies, powers were presented only in absolute units; and in three, only in normalized units.

Only two studies presented results obtained by using methods of nonlinear dynamics—approximate entropy and SD1 and SD2 from the Poincaré plot analysis. An important constraint of time and frequency methods commonly used for HRV analysis is the assumption of linearity in the system under analysis. For example, the spectral analysis assumes that HRV signals are the result of a linear combination of periodic components where certain frequencies are related to the activity of the autonomic sympathetic or parasympathetic nervous system. Consequently, these frequency components are considered all independent of each other, i.e., there are no interactions between the autonomic components. However, it is known that cardiovascular control involves nonlinear interactions between physiological systems, with complex dynamics. Therefore, nonlinear methods appear as complementary and important tools to extract information from HRV series, and we strongly recommend their use in studies of HRV [105,106]. Recently, Sassi et al., (2015), on behalf of the European Heart Rhythm Association, reviewed some of the most used nonlinear approaches and their contributions to HRV understanding [107]. However, it is recognized that no single method (linear or nonlinear) is capable of extracting the full complexity of physiological systems by itself, pointing to the importance of combining multiple HRV parameters to characterize physiological and physiopathological conditions [108].

#### 4.2.4. HRV Correction for HR

The majority of HRV studies, including those on patients with CP reviewed here, have not accounted for the significant correlation between HRV parameters and mean HR. In light of our current knowledge, HRV is primarily HR-dependent in adults [28,29,30,31] and children [109,110], i.e., different HR may exert different impact on HRV and, to some extent, may determine HRV values [32,36,111,112,113,114,115,116,117]. Correction methods were proposed and it was highlighted that the HRV dependence on HR should be removed before drawing conclusions on HRV changes [118]. However, recently, such an approach was discussed, but the authors recommended that any relationships between the prevailing heart period and the HRV parameters should always be formally examined and reported [44]. Researchers and clinicians should also be aware that not all HRV indices are dependent on mean HR [119].

### 4.3. Lack of Methodological Information in Existing Studies on HRV in Pediatric Participants with CP

Controlling all the possible confounding factors and reporting all the methodological aspects in the study is crucial for results interpretation, reproducibility, and translating the findings into the clinical practice. The methodological heterogeneity observed in the reviewed studies and the lack of information provided may account for some controversial results and limit the insights and the comparisons between different studies. Furthermore, it directly impacts health professionals dealing with this population due to uncertainty on how to use these findings in clinical settings. Although HRV provides important clinical markers, it remains underutilized and raises several issues about its clinical applicability [120]. Thus, following the methodological guidelines for RR recording and HRV analysis and properly reporting them is required to increase HRV clinical use by health professionals.

## 5. Conclusions

In summary, pediatric patients with CP presented significantly higher resting HR and a reduced HRV, as well as different responses (or no effect) to selected movement maneuvers compared to typically developed children and decreased values of selected HRV parameters in those dependent on adult assistance for mobility compared to those who are generally independent. Physical exercises positively influence cardiac autonomic regulation by reducing HR and breathing rate and increasing values of selected HRV parameters. However, most HRV studies in this population failed to provide the necessary details concerning data acquisition and HRV measurements, so the experimental design could not be replicated in laboratories or clinical settings. Thus, the lack of details concerning methodological aspects of these studies significantly limits their replication and confidence in interpretation [35]. It is important that authors of future studies on HRV in patients with CP are aware about recent controversies of HRV measurement and follow the recently developed HRV metrics, as well as their physiological interpretation and meaning [121,122,123,124,125,126,127]. Following the current guidelines will help to identify and eliminate most methodological limitations and misinterpretations.

Dan (2017) emphasized that data from studies on the ANS in patients with CP “are necessary to discuss possible implications for clinical practice in terms of diagnosis, outcome” [128] (p. 1). Developing a better understanding of ANS activity in patients with CP is required to better understand cardiovascular diseases in this group of patients. Moreover, studies highlight the importance of non-autonomic influences in HRV [129,130,131], which are also a potential source of important information to understand the impairments in patients with CP and should be taken into account in future studies.

## Figures and Tables

**Table 1:** Baseline characteristics of study participants and details concerning RR interval acquisition.

First Author and Year of Publication	Experimental Group	Control Group	RR Intervals Acquisition				
			Software for RR Intervals Acquisition, Sampling Frequency, and Duration of Recordings	Time of the Day and Room (Lights/Voices/Temperature)	Activities (Sleep Routine, Physical Activities, Meals, Drinks, Using the Toilet before Recordings) and Instructions Given. Time Reported for Rest or Heart Rate Stabilization before Recordings	Respiratory Rate (Breathing Control) during Recordings	Position during Recordings
Park et al., 2002 [2]	12 children with CP (7♂). Age: 6–11 years; 5: quadriplegia, 4: diplegia, 3: hemiplegia	12 normally developed children (7♂). Age: 5–12 years	Software: software developed by the authors Sampling: 1000 Hz Duration: 3 min	Measurements were carried out at about 3:00 pm in a quiet room at room temperature 20–24 °C.	Subjects had a very light lunch. 10 min	Subjects were instructed to breathe with a metronome at 15 breaths/min (0.25 Hz).	Supine, 70° head-up tilt using a tilt table
Yang et al., 2002 [3]	30 children with CP (18♂). Age: 4–10 years; 7: quadriplegia, 23: diplegia	30 age- and sex-matched normally developed children	Software: software developed by one of the authors Sampling: 256 Hz Duration: 288 s	Not reported	Not reported 15 min	Not reported	Supine, head-up tilt (angle not specified)
Ferreira et al., 2011 [7]	90 children with CP (58♂). Age: 3–15 years; 31: quadriplegia, 31: diplegia, 6: hemiplegia	35 individuals matched by age	Software: Electrocardiography (ECG) Holter monitoring (SEER Light, GE Medical Systems, Milwaukee, WI, USA) Sampling: 250 Hz Duration: 24 h	24 h monitoring	Not reported Not reported	Not reported	Not applicable
Zamunér et al., 2011 [5]	12 children with CP (7♂). Age: 4–13 years; 4: quadriplegia, 6: diplegia, 2: hemiplegia	16 children with typical motor development (5♂)	Software: Nerve–Express system software (Heart Rhythm Instruments, Inc., Metuchen, NJ, EUA) Sampling: not reported Duration: 15 min	Not reported	The children and their parents were given instructions to avoid consumption of stimulating beverages, to suspend any major physical activity, to have light meals, and to have a good night’s rest. All children were familiarized with the experimental proceedings during a pilot test conducted a week prior to the study procedures. The children were asked not to talk or to move during data collection. Not reported	The children maintained spontaneous breathing, presenting 10 to 20 breaths per minute.	Supine, standing
Kholod et al., 2013 [8]	26 children with CP (12♂). Age: 8–14 years; 13: quadriplegia, 9: diplegia, 4: hemiplegia, 2: athetoid signs. GMFCS I-V	16 typically developed children (6♂) matched for age	Software: 12-lead digital ECG Holter recorder (DR180 Digital Recorder; NorthEast Monitoring Inc. Maynard, Mass) Sampling: not reported Duration: lack of precise description. The ECG was continuously monitored throughout the test procedure.	All procedures were performed in a quiet room, with the temperature between 21–26°C.	Before data collection, each subject was familiarized with the study protocol. Every attempt was made to control external factors: similar assessment time, restriction of activity, and/or heavy meal prior to the Holter recording. Lack of precise description	Not reported	Supine, during walking
Israeli-Mendlovic et al., 2014 [9]	30 children with CP (17♂). Age: 6–12 years; 25: quadriplegia, 5: dyskinesia. GMFCS IV-V	No control group	Software: Polar Advanced Heart Rate Monitor (RC800CX) Sampling: not reported Duration: supine—10 min, GMFM assessment, rest—5 min, highest activity achieved in the GMFM assessment performed over and over again for 2 min, standing—10 min	All procedures were performed in a quiet room, with the temperature between 21–26 °C.	Before data collection, each subject was familiarized with the study protocol. Lack of precise description	Not reported	Supine, during activities (GMFM assessment), standing
Amichai et al., 2017 [11]	20 children with CP (12♂). Age: 6–11 years; 12: diplegia, 8: hemiplegia. GMFCS I-III	No control group	Software: Polar Advanced Heart Rate Monitor (RC800CX) Sampling: Not reported. Duration: lack of precise description. No information whether 5 min were dedicated for a rest or RR interval recording.	Not reported	The children were asked to sit quietly at rest for 5 min and then to walk on the treadmill. Not reported	Not reported	Sitting, walking
Cohen-Holzer et al., 2017 [13]	24 children with unilateral CP (16♂). Age: 6–10 years; GMFCS I-II	No control group	Software: Polar Advanced Heart Rate Monitor (RS800CX) Sampling: not reported Duration: not reported	Not reported	Not reported Not reported	Not reported	Not reported
Kim et al., 2017 [56]	13 children with CP (8♂) considered as control group. Mean age: 7.5 years (1.9–16.0); GMFCS I-III	CP children were considered the control group for the children with acute brain injury	Software: not reported Sampling: not reported Duration: 5 min	Noise-free environment. Data were collected between 1:00 and 3:00 PM. The room temperature during data collection was 24–26 °C.	Not reported 30 min	Not reported	Supine
Amichai et al., 2019 [12]	20 children with CP (15♂). Age: 6–11 years; 11: diplegia, 7: hemiplegia, 2: quadriplegia. GMFCS I–III	20 typically developed children (14♂) matched for age and gender	Software: Polar Advanced Heart Rate Monitor (RS800CX) Sampling: not reported Duration: HRV data were recorded throughout the entire session.	Not reported	Not reported Not reported	The children were asked to lie down quietly on the back for 5 min, then to sit quietly in a resting state for 5 min, followed by a paced breathing training (15 min). Paced breathing and breathing rate were evaluated using the ProRelax software (ver. 5.1) and a chest belt	Supine (HRV), sitting (HRV and breathing manipulation)
Katz-Leurer et al., 2019 [14]	110 children with CP (66♂). Age: 6–11 years; GMFCS I-V	35 typically developed children matched for age	Software: Polar Advanced Heart Rate Monitor RC800CX Sampling: not reported Duration: 10 min	Testing was performed in the morning hours, in a quiet room with the temperature between 21–26 °C.	The children were asked not to consume a heavy meal, drink caffeinated beverages, or perform physical activities for at least 2 h before testing. Not reported	Not reported	Not reported
Landis et al., 2019 [54]	10 children with CP (4♂). Mean age: 15.5 ± 3.6 years; 4: diplegia, 6: hemiplegia. GMFCS II-III	No control group	Software: Heart rate monitor (name of the software not reported) Sampling: 250 Hz Duration: 5 min; conditioning phase (10 min) divided into two 5 min phases	Not reported	Not reported 5 min	Not reported	Sitting

CP—cerebral palsy; GMFCS—Gross Motor Function Classification System; GMFM—Gross Motor Function Measure; HRV—heart rate variability; ECG—electrocardiography.

**Table 2:** Heart rate variability (HRV) measurement.

First Author and Year of Publication	Software	Artifact Correction	Time Series Length (Time/Beats)	Information about Data Normality	Time Domain Parameters (Units)	Frequency Domain Parameters and Bands (Units)	Frequency Analysis Method with Details	Nonlinear Parameters
Park et al., 2002 [2]	Software developed by the authors.	Modified spatial velocity algorithm to detect QRS peaks. The signals were passed through a band pass filter of 0.1–150 Hz to eliminate unwanted noise signals.	Not reported	Not mentioned	Did not perform time domain analysis.	LF: 0.05–0.15 Hz (ms^2^, nu) HF: 0.15–0.40 Hz (ms^2^, nu) TP (ms^2^) LF/HF	Cubic spline interpolation method. Autoregressive model using the Burg’s maximum entropy method.	Did not perform nonlinear analysis.
Yang et al., 2002 [3]	Software developed by one of the authors	For the RR interval rejection procedure, a temporary mean and the standard deviation of all RR intervals were first calculated as the standard reference. Each RR interval was then validated with respect to this reference. If the standard score of an RR value exceeded 3, it was considered erroneous or non-stationary and was thus rejected. The valid RR values were then resampled and interpolated at the rate of 7.11 Hz to accomplish continuity in the time domain.	288 s/2048 data points	Not mentioned	Did not perform time domain analysis.	LF: 0.04–0.15 Hz (nu) HF: 0.15–0.40 Hz (nu)	Fast Fourier transformation. Resulting power spectrum was corrected for attenuation resulting from the sampling process and the Hamming window.	Did not perform nonlinear analysis.
Ferreira et al., 2011 [7]	Not reported	Data were processed and analyzed using a 250 Hz sampling frequency (GE MARS 7.1 equipment with MARS 7.1; GE Medical System software).	Normal RR intervals over a period of at least 18 h of the analyzable signal were analyzed.	Not mentioned	SDNN (ms) pNN50 (%)	VLF: 0.003–0.04 Hz (ms^2^) LF: 0.04–0.15 Hz (ms^2^) HF: 0.15–0.40 Hz (ms^2^) TP (ms^2^) LF/HF	Not reported	Did not perform nonlinear analysis.
Zamunér et al., 2011 [5]	Nerve–Express system software	Not reported	5 min	Data distribution was tested using the Shapiro–Wilk test, and the normality hypothesis of all variables was rejected.	Did not perform time domain analysis.	LF: 0.04–0.15 Hz (nu) HF: 0.15–0.40 Hz (nu)	Authors reported to select the highest stability section RR intervals and to perform an autoregressive spectral analysis.	Did not perform nonlinear analysis.
Kholod et al., 2013 [8]	NorthEast Monitoring’s Holter LX Enhanced Plus Software (version 5.2 Beta)	RR intervals were visually inspected and then filtered with the HRV software to eliminate undesirable noise or premature beats.	Not reported	Normality distribution checked (method not specified).	SDNN (ms) RMSSD (ms)	Did not perform frequency domain analysis.	Not applicable	Did not perform nonlinear analysis.
Israeli-Mendlovic et al., 2014 [9]	Not reported	Beat intervals were visually inspected and then filtered with the HRV software to eliminate undesirable noise.	Not reported	Not mentioned	SDNN (ms) RMSSD (ms)	LF: 0.04–0.15 Hz (nu) HF: 0.15–0.40 Hz (nu) LF/HF	Not reported	Did not perform nonlinear analysis.
Amichai et al., 2017 [11]	Not reported	The interbeat intervals were visually inspected and filtered with the HRV software to eliminate noise.	Not reported	Not mentioned	SDNN [ms] RMSSD (ms)	LF/HF	Not reported	SD1 (ms) SD2 (ms)
Cohen-Holzer et al., 2017 [13]	Kubios heart rate variability software version 2.0; Biosignal Analysis and Medical Imaging Group	Not reported	Not reported	Not mentioned	SDNN [ms] RMSSD (ms)	Did not perform frequency domain analysis.	Not applicable	Did not perform nonlinear analysis.
Kim et al., 2017 [56]	SA-6000 device (Medicore Co., Seoul, Korea)	Abnormal beats, significant pauses, and areas of artifact were automatically rejected by using a computerized algorithm.	5 min	Not mentioned	SDNN [ms] RMSSD (ms)	LF: 0.04–0.15 Hz (ms^2^, nu) HF: 0.15–0.40 Hz (ms^2^, nu) TP (ms^2^) LF/HF	Fast Fourier transform	ApEn
Amichai et al., 2019 [12]	Not reported	The interbeat intervals were visually inspected and then filtered using the HRV software to eliminate undesirable noise.	5 min	The Kolmogorov–Smirnov test was performed for all outcome measures.	SDNN [ms] RMSSD (ms)	LF: 0.04–0.15 Hz (ms^2^) HF: 0.15–0.40 Hz (ms^2^) LF/HF	Fast Fourier transform	Did not perform nonlinear analysis.
Katz-Leurer et al., 2019 [14]	Not reported	Not reported	Not reported	Not mentioned	mRR (ms) SDNN (ms) RMSSD (ms)	LF/HF	Not reported	Did not perform nonlinear analysis.
Landis et al., 2019 [54]	Not reported	The aim of this study was to generate a method for calculating HRV from ECG waveforms. Preliminary R peak detection and peak correction described with details.	5 min	Not mentioned	avNN (s) RMSSD (ms) SDNN (ms) NN50 (count) pNN50 (%)	LF (RR)* HF (RR) LF/HF (RR) LF/HF (ECG)** Units not reported	Fast Fourier transform	Did not perform nonlinear analysis.

VLF—very low frequency; LF—low frequency; HF—high frequency; TP—total power; nu—normalized units; mRR—mean RR interval; avNN—average NN interval; NN—intervals between normal R-peaks; SDNN—standard deviation of NN intervals; RMSSD—root mean square successive difference; pNN50—percentage of adjacent NN intervals that differ from each other by more than 50 ms; SD1—standard deviation of the distance of each point from the y = x axis, specifies the ellipse’s width; SD2—standard deviation of each point from the y = x + average RR interval, specifies the ellipse’s length; ApEn—approximate entropy; HRV—heart rate variability; QRS peak—represents flow of electrical impulse through the septum and outer ventricles; * and ** from Landis et al., 2019: * (RR)—where R is a point associated with a peak of the QRS complex of the ECG wave and RR is the interval between successive R points; ** (ECG)—Electrocardiogram—which contains the QRS complex.

**Table 3:** Main results and conclusions from included studies.

First Author and Year of Publication	Main Results and Conclusion Related to HRV
Park et al., 2002 [2]	Main results (1) LF/HF ratio was higher in children with CP than in controls. (2) During a head-up tilt, HR, LF, LFnu, and LF/HF increased in the controls, but not in the participants with CP. Conclusions Vagal withdrawal and sympathetic activation, which occur during a head-up tilt position, are not sufficient to overcome the orthostatic stress arising in children with spastic CP.
Yang et al., 2002 [3]	Main results (1) No significant differences were observed between the controls and the children with CP. (2) During a head-up tilt, the controls featured increased LF and LF/HF ratio and decreased HF. The children with CP did not present any differences between the supine position and a head-up tilt. Conclusions The disturbed balance of activity between the sympathetic and parasympathetic nervous system observed in the study might result from the loss of hemispheric influence in patients with CP; however, further investigation is clearly necessary.
Ferreira et al., 2011 [7]	Main results The group of children with CP presented higher HF and LF values and lower LF/HF compared to the controls. Conclusions Individuals with CP present an increased cardiovascular risk, a disturbed sympathovagal balance that could contribute to the salivary secretion alterations observed.
Zamunér et al., 2011 [5]	Main results (1) The control group presented a higher HFnu value and a lower LFnu value compared to the children with CP in the supine position. (2) During standing, the controls featured increased LFnu and decreased HFnu. The children with CP did not present any differences between the supine position and standing. (3) There was a significant correlation between the GMFCS class and the LFnu index, the HFnu index, and the LF/HF ratio. Conclusions Children with CP present lower HRV indices, indicating sympathovagal imbalance. The decrease of HRV in children with CP is related to the motor impairment level.
Kholod et al., 2013 [8]	Main results (1) The children with CP presented higher mean HR and lower time domain values at rest in comparison to the controls. (2) There was no association between HR and HRV and motor performance (GMFM score) in the children with CP. (3) The children with CP at different disability levels showed similar HRV values. Conclusions Among children with CP, the cardiac autonomic mechanism is less efficient at rest and less adaptive to exercise and activity as compared to typically developed children.
Israeli-Mendlovic et al., 2014 [9]	Main results (1) The children with GMFCS IV presented increased HR and reduced HRV during the GMFM assessment, the repeated task, and during passive standing. No such effect was noted among the children with GMFCS V. (2) No significant differences were noted in the HR or HRV parameters based on activity level. Conclusions HR autonomic regulation system has an opportunity to be influenced by training in children with CP GMFCS IV.
Amichai et al., 2017 [11]	Main results (1) HR increased during the last stage of the treadmill test compared with the rest. The RMSSD was reduced during the last two minutes of the treadmill test compared with the rest. (2) The HR and RMSSD mean value at the second minute post-test were not significantly different from the pre-treadmill rest value. Conclusions Further studies are needed to assess the possible influence of exercise protocols on the cardiac autonomic system.
Cohen-Holzer et al., 2017 [13]	Main results Significant reduction in HR and an increase in RMSSD 3 months post-intervention.Conclusions An intensive hybrid program (10 days, 6 h per day) effectively improved the cardiac autonomic regulation system.
Kim et al., 2017 [56]	Main results (1) There were significant differences between the patients with CP and acute brain injury in the mean HR, RMSSD, and all indices of the frequency domain analysis. (2) The mean HR, normalized LF, and the LF/HF ratio decreased in the group of children with CP. Conclusions Patients with acute brain injury have higher sympathetic excitatory activity and more dominant sympathetic power than the parasympathetic power compared to the control group. Presence of paroxysmal sympathetic hyperactivity symptoms were noted among the children with acute brain injury compared to the age- and sex-matched control group of children with CP.
Amichai et al., 2019 [12]	Main results (1) Children with CP have lower spirometry and HRV values at rest compared to TD children. (1) The mean reduction of the breathing rate during paced breathing among the children with CP was significantly smaller. Conclusions Children with CP have the ability to perform paced breathing training in order to influence their respiratory rate. HRV parameters increase during a paced breathing practice in children with CP, showing an impact on the cardiac autonomic control system.
Katz-Leurer et al., 2019 [14]	Main results (1) There were significant differences in all HRV measures between groups, with significantly lower mRR, SDNN, and RMSSD values, and higher LF/HF values in the children with CP versus the controls. (2) Significant differences between the patients with five different GMFCS levels were noted in all HRV measures. Conclusions Assessing appropriate protocols for improving autonomic regulation in children with CP is the next step needed.
Landis et al., 2019 [54]	Main results The authors presented an active video game data collection protocol and a methodology to calculate HRV from the ECG data obtained via an HR monitor. Conclusions The proposed methodology allows to extract RR intervals and HRV measures from ECG waveforms during gaming physical activities in youths with CP. The method is currently tailored towards active video game sessions in a specific game, but could easily be adapted to other protocols and ECG devices for future experiments.

CP—cerebral palsy; TD—typically developed; GMFCS—Gross Motor Function Classification System; GMFM—Gross Motor Function Measure; HR—heart rate; HRV—heart rate variability; LF—low frequency; HF—high frequency; nu—normalized units; mRR—mean RR interval; SDNN—standard deviation of NN intervals; RMSSD—root mean square successive difference; ECG—electrocardiography.

## Supplementary Materials

The following are available online at https://www.mdpi.com/2077-0383/9/4/1141/s1, Table S1: Results for heart rate (HR) and time domain HRV parameters, Table S2: Results for frequency domain and nonlinear HRV parameters.

## References

[1] Rosenbaum P., Paneth N., Leviton A., Goldstein M., Bax M., Damiano D., Dan B., Jacobsson B. (2007). A report: The definition and classification of cerebral palsy April 2006. Dev. Med. Child Neurol. Suppl..

[2] Park E.S., Park C.I., Cho S.-R., Lee J.-W., Kim E.J. (2002). Assessment of Autonomic Nervous System with Analysis of Heart Rate Variability in Children with Spastic Cerebral Palsy. Yonsei Med J..

[3] Yang T.F., Chan R.C., Kao C.L., Chiu J.W., Liu T.J., Kao N.T., Kuo T.B.J. (2002). Power Spectrum Analysis of Heart Rate Variability for Cerebral Palsy Patients. Am. J. Phys. Med. Rehabil..

[4] Kerppers I.I., Arisawa E.A.L., Oliveira L.V.F., Sampaio L.M.M., Oliveira C.S. (2009). Heart rate variability in individuals with cerebral palsy. Arch. Med. Sci..

[5] Zamunér A.R., Cunha A.B., Da Silva E., Negri A.P., Tudella E., Moreno M.A. (2011). The influence of motor impairment on autonomic heart rate modulation among children with cerebral palsy. Res. Dev. Disabil..

[6] Pastore C.A., Samesima N., Imada R., Reis M., Santos M.T.B.R., Ferreira M.C., Grupi C., Fumagalli F., Wagenführ J., Chammas M. (2011). Characterization of the electrocardiographic pattern of individuals with cerebral palsy. J. Electrocardiol..

[7] Ferreira M., Pastore C.A., Imada R., Guare R.O., Leite M., Poyares D., Santos M.T.B.R. (2011). Autonomic nervous system in individuals with cerebral palsy: A controlled study. J. Oral Pathol. Med..

[8] Kholod H., Jamil A., Katz-Leurer M. (2013). The associations between motor ability, walking activity and heart rate and heart rate variability parameters among children with cerebral palsy and typically developed controls. Neurorehabilitation.

[9] Israeli-Mendlovic H., Mendlovic J., Katz-Leurer M. (2014). Heart rate and heart rate variability parameters at rest, during activity and passive standing among children with cerebral palsy GMFCS IV–V. Dev. Neurorehabilit..

[10] Amichai T., Katz-Leurer M. (2014). Heart rate variability in children with cerebral palsy: Review of the literature and meta-analysis. Neurorehabilitation.

[11] Amichai T., Eylon S., Dor-Haim H., Berger I., Katz-Leurer M. (2017). Cardiac Autonomic System Response to Submaximal Test in Children with Cerebral Palsy. Pediatr. Phys. Ther..

[12] Amichai T., Eylon S., Berger I., Katz-Leurer M. (2018). The impact of breathing rate on the cardiac autonomic dynamics among children with cerebral palsy compared to typically developed controls. Dev. Neurorehabilit..

[13] Cohen-Holzer M., Sorek G., Schweizer M., Katz-Leurer M. (2017). The influence of a constraint and bimanual training program using a variety of modalities on endurance and on the cardiac autonomic regulation system of children with unilateral cerebral palsy: A self-control clinical trial. Neurorehabilitation.

[14] Katz-Leurer M., Amichai T. (2019). Heart rate variability in children with cerebral palsy. Dev. Med. Child Neurol..

[15] Samuels M.A. (2007). The Brain–Heart Connection. Circulation.

[16] Yiallourou S.R., Witcombe N.B., Sands S., Walker A.M., Horne R.S. (2013). The development of autonomic cardiovascular control is altered by preterm birth. Early Hum. Dev..

[17] Sender N.S., Govindan R.B., Sulemanji M., Al-Shargabi T., Lenin R.B., Eksioglu Y.Z., Du Plessis A.J. (2014). Effects of regional brain injury on the newborn autonomic nervous system. Early Hum. Dev..

[18] Ardell J.L., Andresen M.C., Armour J.A., Billman G.E., Chen P., Foreman R.D., Herring N., O’Leary D.S., Sabbah H.N., Schultz H.D. (2016). Translational neurocardiology: Preclinical models and cardioneural integrative aspects. J. Physiol..

[19] Prathep S., Sharma D., Hallman M., Joffe A., Krishnamoorthy V., Mackensen G.B., Vavilala M.S. (2014). Preliminary Report on Cardiac Dysfunction after Isolated Traumatic Brain Injury. Crit. Care Med..

[20] Grunsfeld A., Fletcher J.J., Nathan B.R. (2005). Cardiopulmonary complications of brain injury. Curr. Neurol. Neurosci. Rep..

[21] Van Der Bilt I., Hasan D., Vandertop W.P., Wilde A.A., Algra A., Visser F.C., Rinkel G.J. (2009). Impact of cardiac complications on outcome after aneurysmal subarachnoid hemorrhage: A meta-analysis. Neurology.

[22] Chen Z., Venkat P., Seyfried D., Chopp M., Yan T., Chen J. (2017). Brain–Heart Interaction. Circ. Res..

[23] Ryan J.M., Crowley V.E., Hensey O., Broderick J., McGahey A., Gormley J. (2014). Habitual physical activity and cardiometabolic risk factors in adults with cerebral palsy. Res. Dev. Disabil..

[24] Ryan J.M., Hensey O., McLoughlin B., Lyons A., Gormley J. (2014). Reduced Moderate-to-Vigorous Physical Activity and Increased Sedentary Behavior Are Associated with Elevated Blood Pressure Values in Children with Cerebral Palsy. Phys. Ther..

[25] Task Force of the European Society of Cardiology and the North American Society of Pacing and Electrophysiology (1996). Heart rate variability: Standards of measurement, physiological interpretation and clinical use. Circulation.

[26] Shaffer F., McCraty R., Zerr C.L. (2014). A healthy heart is not a metronome: An integrative review of the heart’s anatomy and heart rate variability. Front. Psychol..

[27] Peltola M.A. (2012). Role of Editing of R–R Intervals in the Analysis of Heart Rate Variability. Front. Physiol..

[28] Sacha J. (2013). Why should one normalize heart rate variability with respect to average heart rate. Front. Physiol..

[29] Sacha J. (2014). Interaction between Heart Rate and Heart Rate Variability. Ann. Noninvasive Electrocardiol..

[30] Sacha J. (2014). Heart rate contribution to the clinical value of heart rate variability. Kardiol. Pol..

[31] Sacha J. (2014). Interplay between heart rate and its variability: A prognostic game. Front. Physiol..

[32] Billman G.E. (2013). The effect of heart rate on the heart rate variability response to autonomic interventions. Front. Physiol..

[33] Heathers J. (2014). Everything Hertz: Methodological issues in short-term frequency-domain HRV. Front. Physiol..

[34] Quintana D.S., Heathers J. (2014). Considerations in the assessment of heart rate variability in biobehavioral research. Front. Psychol..

[35] Quintana D.S., Alvares G.A., Heathers J.A.J. (2016). Guidelines for Reporting Articles on Psychiatry and Heart rate variability (GRAPH): Recommendations to advance research communication. Transl. Psychiatry.

[36] Billman G.E., Huikuri H.V., Sacha J., Trimmel K. (2015). An introduction to heart rate variability: Methodological considerations and clinical applications. Front. Physiol..

[37] Shaffer F., Ginsberg J. (2017). An Overview of Heart Rate Variability Metrics and Norms. Front. Public Health.

[38] Ernst G. (2017). Heart-Rate Variability—More than Heart Beats?. Front. Public Health.

[39] Ernst G. (2017). Hidden Signals—The History and Methods of Heart Rate Variability. Front. Public Health.

[40] Laborde S., Mosley E., Thayer J.F. (2017). Heart Rate Variability and Cardiac Vagal Tone in Psychophysiological Research – Recommendations for Experiment Planning, Data Analysis, and Data Reporting. Front. Psychol..

[41] Singh N., Moneghetti K.J., Christle J.W., Hadley D., Plews D., Froelicher V. (2018). Heart Rate Variability: An Old Metric with New Meaning in the Era of using mHealth Technologies for Health and Exercise Training Guidance. Part One: Physiology and Methods. Arrhythmia Electrophysiol. Rev..

[42] Singh N., Moneghetti K.J., Christle J.W., Hadley D., Froelicher V., Plews D. (2018). Heart Rate Variability: An Old Metric with New Meaning in the Era of Using mHealth technologies for Health and Exercise Training Guidance. Part Two: Prognosis and Training. Arrhythmia Electrophysiol. Rev..

[43] Hayano J., Yuda E. (2019). Pitfalls of assessment of autonomic function by heart rate variability. J. Physiol. Anthr..

[44] De Geus E.J.C.N., Gianaros P.J., Brindle R.C., Jennings J.R., Berntson G.G. (2018). Should heart rate variability be “corrected” for heart rate? Biological, quantitative, and interpretive considerations. Psychophysiology.

[45] Li K., Rüdiger H., Ziemssen F. (2019). Spectral Analysis of Heart Rate Variability: Time Window Matters. Front. Neurol..

[46] Malik M., Hnatkova K., Huikuri H.V., Lombardi F., Schmid R.M., Zabel M. (2019). CrossTalk proposal: Heart rate variability is a valid measure of cardiac autonomic responsiveness. J. Physiol..

[47] Kemper K.J., Hamilton C., Atkinson M. (2007). Heart Rate Variability: Impact of Differences in Outlier Identification and Management Strategies on Common Measures in Three Clinical Populations. Pediatr. Res..

[48] Thayer J.F., Yamamoto S.S., Brosschot J. (2010). The relationship of autonomic imbalance, heart rate variability and cardiovascular disease risk factors. Int. J. Cardiol..

[49] Xhyheri B., Manfrini O., Mazzolini M., Pizzi C., Bugiardini R. (2012). Heart Rate Variability Today. Prog. Cardiovasc. Dis..

[50] Billman G.E. (2011). Heart Rate Variability – A Historical Perspective. Front. Physiol..

[51] Akinci A., Baykal E., Akinci A., Celiker A., Tezic T. (1993). Heart rate variability in diabetic children: Sensitivity of the time- and frequency-domain methods. Pediatr. Cardiol..

[52] Chessa M., Butera G., Lanza G.A., Bossone E., Delogu A., De Rosa G., Marietti G., Rosti L., Carminati M. (2002). Role of Heart Rate Variability in the Early Diagnosis of Diabetic Autonomic Neuropathy in Children. Herz.

[53] Taşçılar M.E., Yokuşoğlu M., Boyraz M., Baysan O., Koz C., Dündaröz R. (2011). Cardiac Autonomic Functions in Obese Children. J. Clin. Res. Pediatr. Endocrinol..

[54] Landis C., O’Neil M.E., Finnegan A., Shewokis P.A. (2019). Calculating Heart Rate Variability from ECG Data from Youth with Cerebral Palsy During Active Video Game Sessions. J. Vis. Exp..

[55] Bjelakovic B., Ilic S., Dimitrijevic L., Milovanović B., Kostic G., Bjelakovic L., Lukic S. (2010). Heart rate variability in infants with central coordination disturbance. Early Hum. Dev..

[56] Kim S.W., Jeon H.R., Kim J., Kim Y. (2017). Heart Rate Variability Among Children With Acquired Brain Injury. Ann. Phys. Rehabil. Med..

[57] Liberati A., Altman U.G., Tetzlaff J., Mulrow C., Gøtzsche P.C., Ioannidis J.P., Clarke M., Devereaux P.J., Kleijnen J., Moher D. (2009). The PRISMA statement for reporting systematic reviews and meta-analyses of studies that evaluate healthcare interventions: Explanation and elaboration. BMJ.

[58] Moher D., Liberati A., Tetzlaff J., Altman D.G., PRISMA Group (2009). Preferred Reporting Items for Systematic Reviews and Meta-Analyses: The PRISMA Statement. PLoS Med..

[59] Durufle-Tapin A., Colin A., Nicolas B., Lebreton C., Dauvergne F., Gallien P. (2014). Analysis of the medical causes of death in cerebral palsy. Ann. Phys. Rehabil. Med..

[60] Ryan J.M., Peterson M.D., Ryan N., Smith K.J., O’Connell N.E., Liverani S., Anokye N., Victor C., Allen E. (2019). Mortality due to cardiovascular disease, respiratory disease, and cancer in adults with cerebral palsy. Dev. Med. Child Neurol..

[61] Fatisson J., Oswald V., LaLonde F. (2016). Influence Diagram of Physiological and Environmental Factors Affecting Heart Rate Variability: An Extended Literature Overview. Hear. Int..

[62] Scheck S.M., Pannek K., Raffelt D., Fiori S., Boyd R.N., Rose S. (2015). Structural connectivity of the anterior cingulate in children with unilateral cerebral palsy due to white matter lesions. NeuroImage Clin..

[63] Carnevali L., Koenig J., Sgoifo A., Ottaviani C. (2018). Autonomic and Brain Morphological Predictors of Stress Resilience. Front. Mol. Neurosci..

[64] Thayer J.F., Koenig J. (2019). Resting Cerebral Blood Flow and Ethnic Differences in Heart Rate Variability: Links to Self-Reports of Affect and Affect Regulation. NeuroImage.

[65] Chang C., Metzger C.D., Glover G.H., Duyn J.H., Heinze H.-J., Walter M. (2012). Association between heart rate variability and fluctuations in resting-state functional connectivity. NeuroImage.

[66] Pannek K., Boyd R.N., Fiori S., Guzzetta A., Rose S. (2014). Assessment of the structural brain network reveals altered connectivity in children with unilateral cerebral palsy due to periventricular white matter lesions. NeuroImage: Clin..

[67] Veijalainen A., Haapala E.A., Väistö J., Leppänen M.H., Lintu N., Tompuri T., Seppälä S., Ekelund U., Tarvainen M.P., Westgate K. (2019). Associations of physical activity, sedentary time, and cardiorespiratory fitness with heart rate variability in 6- to 9-year-old children: The PANIC study. Eur. J. Appl. Physiol..

[68] Verschuren O., Peterson M.D., Balemans A.C., Hurvitz E.A. (2016). Exercise and physical activity recommendations for people with cerebral palsy. Dev. Med. Child Neurol..

[69] Maltais D.B., Pritchard-Wiart L., Fowler E., Verschuren O., Damiano D.L. (2014). Health-related physical fitness for children with cerebral palsy. J. Child Neurol..

[70] Keawutan P., Bell K., Oftedal S., Ware R.S., Stevenson R.D., Davies P.S.W., Boyd R.N. (2017). Longitudinal physical activity and sedentary behaviour in preschool-aged children with cerebral palsy across all functional levels. Dev. Med. Child Neurol..

[71] Vila X.A., Lado M.J., Cuesta-Morales P. (2019). Evidence Based Recommendations for Designing Heart Rate Variability Studies. J. Med. Syst..

[72] Quintana D.S. (2016). Statistical considerations for reporting and planning heart rate variability case-control studies. Psychophysiol..

[73] Lachenbruch P.A., Cohen J. (1988). Statistical Power Analysis for the Behavioral Sciences.

[74] Jeyhani V., Mahdiani S., Peltokangas M., Vehkaoja A. Comparison of HRV parameters derived from photoplethysmography and electrocardiography signals. Proceedings of the 37th Annual International Conference of the IEEE Engineering in Medicine and Biology Society.

[75] Weippert M., Kumar M., Kreuzfeld S., Arndt D., Rieger A., Stoll R. (2010). Comparison of three mobile devices for measuring R–R intervals and heart rate variability: Polar S810i, Suunto t6 and an ambulatory ECG system. Eur. J. Appl. Physiol..

[76] Vasconcellos F.V., Seabra A., Cunha F., Montenegro R.A., Bouskela E., Farinatti P.T.V. (2015). Heart rate variability assessment with fingertip photoplethysmography and polar RS800cx as compared with electrocardiography in obese adolescents. Blood Press. Monit..

[77] Pinheiro N., Couceiro R., Henriques J., Muehlsteff J., Quintal I., Gonçalves L., Carvalho P. Can PPG be used for HRV analysis?. Proceedings of the 38th Annual International Conference of the IEEE Engineering in Medicine and Biology Society (EMBC).

[78] Gilgen-Ammann R., Schweizer T., Wyss T. (2019). RR interval signal quality of a heart rate monitor and an ECG Holter at rest and during exercise. Graefe’s Arch. Clin. Exp. Ophthalmol..

[79] Williams D.P., Jarczok M.N., Ellis R.J., Hillecke T.K., Thayer J.F., Koenig J. (2016). Two-week test-retest reliability of the Polar ^®^ RS800CX ™ to record heart rate variability. Clin. Physiol. Funct. Imaging.

[80] Quintana D.S., Heathers J., Kemp A.H. (2012). On the validity of using the Polar RS800 heart rate monitor for heart rate variability research. Graefe’s Arch. Clin. Exp. Ophthalmol..

[81] Merri M., Farden D.C., Mottley J.G., Titlebaum E.L. (1990). Sampling frequency of the electrocardiogram for spectral analysis of the heart rate variability. IEEE Trans. Biomed. Eng..

[82] Patel K., Rössler A., Lackner H.K., Trozic I., Laing C., Lorr D., Green D.A., Hinghofer-Szalkay H., Goswami N. (2016). Effect of postural changes on cardiovascular parameters across gender. Medicine.

[83] Krejčí J., Botek M., Mc Kune A. (2018). Stabilization period before capturing an ultra-short vagal index can be shortened to 60 s in endurance athletes and to 90 s in university students. PLoS ONE.

[84] Hirsch J.A., Bishop B. (1981). Respiratory sinus arrhythmia in humans: How breathing pattern modulates heart rate. Am. J. Physiol. Heart Circ. Physiol..

[85] Brown T.E., Beightol L.A., Koh J., Eckberg D.L. (1993). Important influence of respiration on human R-R interval power spectra is largely ignored. J. Appl. Physiol..

[86] Fleming S., Thompson M., Stevens R., Heneghan C., Plüddemann A., Maconochie I., Tarassenko L., Mant D. (2011). Normal ranges of heart rate and respiratory rate in children from birth to 18 years of age: A systematic review of observational studies. Lancet.

[87] Gąsior J.S., Sacha J., Jeleń P., Zielinski J., Przybylski J. (2016). Heart Rate and Respiratory Rate Influence on Heart Rate Variability Repeatability: Effects of the Correction for the Prevailing Heart Rate. Front. Physiol..

[88] Sinnecker D., Dommasch M., Barthel P., Müller A., Dirschinger R.J., Hapfelmeier A., Huster K.M., Laugwitz K.-L., Malik M., Schmidt G. (2014). Assessment of mean respiratory rate from ECG recordings for risk stratification after myocardial infarction. J. Electrocardiol..

[89] Nielsen L.G., Folkestad L., Brodersen J.B., Brabrand M. (2015). Inter-Observer Agreement in Measuring Respiratory Rate. PLoS ONE.

[90] Sandercock G.R., Gladwell V., Dawson S., Nunan D., Brodie D., Beneke R. (2008). Association between RR interval and high-frequency heart rate variability acquired during short-term, resting recordings with free and paced breathing. Physiol. Meas..

[91] Kobayashi H. (2009). Does Paced Breathing Improve the Reproducibility of Heart Rate Variability Measurements?. J. Physiol. Anthr..

[92] Frederiks J., Swenne C.A., TenVoorde B.J., Honzikova N., Levert J.V., Maan A.C., Schalij M.J., Bruschke A.V. (2000). The importance of high-frequency paced breathing in spectral baroreflex sensitivity assessment. J. Hypertens..

[93] Faes L., Nollo G., Porta A. (2011). Information Domain Approach to the Investigation of Cardio-Vascular, Cardio-Pulmonary, and Vasculo-Pulmonary Causal Couplings. Front. Physiol..

[94] Wang Y.-P., Kuo T.B.J., Lai C.-T., Chu J.-W., Yang C.C.H. (2013). Effects of respiratory time ratio on heart rate variability and spontaneous baroreflex sensitivity. J. Appl. Physiol..

[95] Shaffer F., Combatalade D.C. (2013). Don’t Add or Miss a Beat: A Guide to Cleaner Heart Rate Variability Recordings. Biofeedback.

[96] Soler A.I.R., Silva L.E.V., Fazan R., Murta L.O., Junior L.O.M. (2018). The impact of artifact correction methods of RR series on heart rate variability parameters. J. Appl. Physiol..

[97] Clifford G., Tarassenko L. (2005). Quantifying Errors in Spectral Estimates of HRV Due to Beat Replacement and Resampling. IEEE Trans. Biomed. Eng..

[98] Jarrin D.C., McGrath J.J., Giovanniello S., Poirier P., Lambert M. (2012). Measurement fidelity of heart rate variability signal processing: The devil is in the details. Int. J. Psychophysiol..

[99] Peng C.-K., Havlin S., Stanley H.E., Goldberger A.L. (1995). Quantification of scaling exponents and crossover phenomena in nonstationary heartbeat time series. Chaos.

[100] Porta A., Guzzetti S., Montano N., Furlan R., Pagani M., Malliani A., Cerutti S. (2001). Entropy, entropy rate, and pattern classification as tools to typify complexity in short heart period variability series. IEEE Trans. Biomed. Eng..

[101] Costa M., Goldberger A.L., Peng C.-K. (2005). Multiscale entropy analysis of biological signals. Phys. Rev. E.

[102] Heathers J. (2013). The last word. Exp. Physiol..

[103] Pichon A., Roulaud M., Antoine-Jonville S., De Bisschop C., Denjean A. (2006). Spectral analysis of heart rate variability: Interchangeability between autoregressive analysis and fast Fourier transform. J. Electrocardiol..

[104] Montano N., Ruscone T.G., Porta A., Lombardi F., Pagani M., Malliani A. (1994). Power spectrum analysis of heart rate variability to assess the changes in sympathovagal balance during graded orthostatic tilt. Circulation.

[105] Acharya U.R., Joseph K.P., Kannathal N., Lim C.M., Suri J.S. (2006). Heart rate variability: A review. Med Boil. Eng..

[106] Huikuri H.V., Perkiömäki J.S., Maestri R., Pinna G.D. (2009). Clinical impact of evaluation of cardiovascular control by novel methods of heart rate dynamics. Philos. Trans. R. Soc. A Math. Phys. Eng. Sci..

[107] Sassi R., Cerutti S., Lombardi F., Malik M., Huikuri H.V., Peng C.-K., Schmidt G., Yamamoto Y., Gorenek B., Lip G.Y. (2015). Advances in heart rate variability signal analysis: Joint position statement by the e-Cardiology ESC Working Group and the European Heart Rhythm Association co-endorsed by the Asia Pacific Heart Rhythm Society. Europace.

[108] Voss A., Schulz S., Schroeder R., Baumert M., Caminal P. (2008). Methods derived from nonlinear dynamics for analysing heart rate variability. Philos. Trans. R. Soc. A Math. Phys. Eng. Sci..

[109] Gąsior J.S., Sacha J., Jeleń P., Pawłowski M., Werner B., Dąbrowski M.J. (2015). Interaction Between Heart Rate Variability and Heart Rate in Pediatric Population. Front. Physiol..

[110] Gąsior J.S., Sacha J., Pawłowski M., Zieliński J., Jeleń P., Tomik A., Ksiazczyk T., Werner B., Dąbrowski M.J. (2018). Normative Values for Heart Rate Variability Parameters in School-Aged Children: Simple Approach Considering Differences in Average Heart Rate. Front. Physiol..

[111] Sacha J., Grzeszczak W. (2001). Left ventricular mass index determines variability of the sinus rhythm in essential hypertension. New insight into heart period fluctuations via corrected spectral analysis. Folia Cardiol..

[112] Sacha J., Pluta W. (2005). Different methods of heart rate variability analysis reveal different correlations of heart rate variability spectrum with average heart rate. J. Electrocardiol..

[113] Sacha J., Pluta W. (2008). Alterations of an average heart rate change heart rate variability due to mathematical reasons. Int. J. Cardiol..

[114] Sacha J., Barabach S., Statkiewicz-Barabach G., Sacha K., Müller A., Piskorski J., Barthel P., Schmidt G. (2013). How to select patients who will not benefit from ICD therapy by using heart rate and its variability?. Int. J. Cardiol..

[115] Sacha J., Barabach S., Statkiewicz-Barabach G., Sacha K., Müller A., Piskorski J., Barthel P., Schmidt G. (2013). How to strengthen or weaken the HRV dependence on heart rate — Description of the method and its perspectives. Int. J. Cardiol..

[116] Sacha J., Sobon J., Sacha K., Barabach S. (2013). Heart rate impact on the reproducibility of heart rate variability analysis. Int. J. Cardiol..

[117] Sacha J., Barabach S., Statkiewicz-Barabach G., Sacha K., Müller A., Piskorski J., Barthel P., Schmidt G. (2014). Gender differences in the interaction between heart rate and its variability — How to use it to improve the prognostic power of heart rate variability. Int. J. Cardiol..

[118] Monfredi O., Lyashkov A.E., Johnsen A.-B., Inada S., Schneider H., Wang R., Nirmalan M., Wisløff U., Maltsev V.A., Lakatta E.G. (2014). Biophysical Characterization of the Underappreciated and Important Relationship Between Heart Rate Variability and Heart Rate. Hypertension.

[119] Silva L.E.V., Salgado H.C., Fazan R. (2017). Mean Heart Rate Level Does Not Affect All Heart Rate Variability Indices. Hypertens..

[120] Huikuri H.V., Mäkikallio T., Airaksinen J., Mitrani R., Castellanos A., Myerburg R.J. (1999). Measurement of heart rate variability: A clinical tool or a research toy?. J. Am. Coll. Cardiol..

[121] Eckberg D.L. (1997). Sympathovagal Balance. Circulation.

[122] Moak J.P., Goldstein D.S., Eldadah B.A., Saleem A., Holmes C., Pechnik S., Sharabi Y. (2007). Supine low-frequency power of heart rate variability reflects baroreflex function, not cardiac sympathetic innervation. Hear. Rhythm..

[123] Goldstein D.S., Bentho O., Park M.-Y., Sharabi Y. (2011). Low-frequency power of heart rate variability is not a measure of cardiac sympathetic tone but may be a measure of modulation of cardiac autonomic outflows by baroreflexes. Exp. Physiol..

[124] Rahman F., Pechnik S., Gross D.J., Sewell L., Goldstein D.S. (2011). Low frequency power of heart rate variability reflects baroreflex function, not cardiac sympathetic innervation. Clin. Auton. Res..

[125] Heathers J. (2012). Sympathovagal balance from heart rate variability: An obituary. Exp. Physiol..

[126] Billman G.E. (2013). The LF/HF ratio does not accurately measure cardiac sympatho-vagal balance. Front. Physiol..

[127] Del Paso G.A.R., Langewitz W., Mulder L.J.M., Van Roon A., Duschek S. (2013). The utility of low frequency heart rate variability as an index of sympathetic cardiac tone: A review with emphasis on a reanalysis of previous studies. Psychophysiology.

[128] Dan B. (2017). Understanding the autonomic nervous system in cerebral palsy. Dev. Med. Child Neurol..

[129] Yaniv Y., Ahmet I., Liu J., Lyashkov A.E., Guiriba T.-R., Okamoto Y., Ziman B.D., Lakatta E.G. (2014). Synchronization of sinoatrial node pacemaker cell clocks and its autonomic modulation impart complexity to heart beating intervals. Hear. Rhythm..

[130] Costa M.D., Davis R.B., Goldberger A.L. (2017). Heart Rate Fragmentation: A Symbolic Dynamical Approach. Front. Physiol..

[131] Costa M.D., Davis R.B., Goldberger A.L. (2017). Heart Rate Fragmentation: A New Approach to the Analysis of Cardiac Interbeat Interval Dynamics. Front. Physiol..

