# Supplementary material for: Heart Rate Variability in Children and Adolescents with Cerebral Palsy—A Systematic Literature Review
Source: J Clin Med. 2020 Apr 16;9(4):1141. doi: 10.3390/jcm9041141 (PMC7230809; doi:10.3390/jcm9041141)
Supplement: Supplementary file 1 [file jcm-09-01141-s001.pdf]

Journal of
Clinical Medicine

Table S1. Results for heart rate (HR) and time domain HRV parameters.

First author and year of publication	HR	Mean RR interval	SDNN	RMSSD	pNN50
Park et al. 2002 [2]	Supine → 70º head-up tilt:<br>CP: 97.7 ± 8.3 bpm → 99.4 ± 10.5 bpm<br>CG: 95.5 ± 12.3 bpm → 100.1 ± 12.8 bpm	NDR	NDR	NDR	NDR
Yang et al. 2002 [3]	NDR	NDR	NDR	NDR	NDR
Ferreira et al. 2011 [7]	CP: 96.9 ± 10.8 bpm<br>CG: 92.5 ± 11.5 bpm	NDR	CP: 107.6 ± 38.1 ms<br>CG: 121.1 ± 33.9 ms	NDR	CP: 15.1 ± 12.2 %<br>CG: 17.0 ± 9.3 %
Zamunér et al. 2011 [5]	NDR	NDR	NDR	NDR	NDR
Kholod et al., 2013 [8]	CP: 98.5 ± 13.9 bpm<br>GMFCS I-III: 97.4 ± 12.9 bpm<br>GMFCS IV-V: 99.6 ± 13.5 bpm<br>CG: 83.0 ± 11.6 bpm<br><br>Rest pre walking → walking → rest post walking:<br>CP (N=13): 97.4 ± 12.9 bpm → 107.9 ± 21.3 bpm → 107.5 ± 17.8 bpm<br>CG (N=16): 83.0 ± 11.6 bpm → 105.4 ± 9.9 bpm → 86.1 ± 14.2 bpm	NDR	CP: 61.0 ± 20.8 ms<br>GMFCS I-III: 58.6 ± 23.4 ms<br>GMFCS IV-V: 63.4 ± 18.4 ms<br>CG: 86.2 ± 29.6 ms<br><br>Rest pre walking → walking → rest post walking:<br>CP: 58.6 ± 23.4 ms → 48.9 ± 19.4 ms → 51.8 ± 17.4 ms<br>CG: 86.2 ± 29.6 ms → 57.1 ± 8.6 ms → 69.2 ± 15.1 ms	CP: 52.1 ± 29.2 ms<br>CP GMFCS I-III: 47.6 ± 28.6 ms<br>CP GMFCS IV-V: 56.3 ± 30.6 ms<br>CG: 87.0 ± 39.8 ms<br><br>Rest pre walking → walking → rest post walking: CP: 47.6 ± 28.6 ms → 38.9 ± 19.8 ms → 37.4 ± 22.1 ms<br>CG: 87.0 ± 39.8 ms → 33.2 ± 27.7 ms → 77.5 ± 28.9 ms	NDR
Israeli-Mendlovic et al., 2014 [9]	Rest → GMFM → activity → standing:<br>CP all: 100.7 ± 10.2 bpm → 110.7 ± 15.1 bpm → 127.3 ± 21.9 bpm → 108.6 ± 12.6 bpm<br>GMFCS IV (N=17): 101.8 ± 10.3 bpm → 117.5 ± 12.1 bpm → 139.7 ± 17.4 bpm → 113.1 ± 11.3 bpm	NDR	Rest → GMFM → activity → standing:<br>CP all: 62.6 ± 20.7 ms → 62.9 ± 33.1 ms → 42.1 ± 34.4 ms → 53.5 ± 23.1 ms<br>GMFCS IV: 63.2 ± 15.5 ms → 62.1 ± 36.6 ms → 25.0 ± 16.0 ms → 43.3 ± 16.0 ms	Rest → GMFM → activity → standing:<br>CP all: 44.5 ± 22.1 ms → 33.3 ± 24.9 ms → 20.5 ± 16.5 ms → 29.0 ± 19.0 ms<br>GMFCS IV: 46.1 ± 19.4 ms → 26.7 ± 15.7 ms → 13.9 ± 13.0 ms → 23.4 ± 11.7 ms	NDR

	GMFCS V (N=13): 99.0 ± 10.4 bpm → 100.8 ± 14.6 bpm → 107.0 ± 11.4 bpm → 100.6 ± 10.6 bpm		GMFCS V: 61.6 ± 28.4 ms → 64.0 ± 29.4 ms → 65.5 ± 37.6 ms → 67.6 ± 25.2 ms	GMFCS V: 41.9 ± 27.3 ms → 43.6 ± 33.0 ms → 30.8 ± 16.9 ms → 37.6 ± 25.4 ms	
Amichai et al., 2017 [11]	Rest → last test stage → 1 and 2 min posttest: 93.9 ± 17.1 bpm (86.1 – 102.9) → 140.8 bpm (133.1–148.4) → 127.3 bpm (119.0–135.6) → 102.1 bpm (91.7–112.4)	NDR	Rest: 63.1 ± 32.7 ms	Rest → last test stage → 1 and 2 min posttest: 52.7 ± 36.4 ms (34.3–70.6) → 11.2 ms (5.3–17.0) → 19.7 ms (11.9–27.5) → 33.4 ms (22.0–44.8)	NDR
Cohen-Holzer et al., 2017 [13]	Pre → post intervention → 3-month post intervention: 94 bpm (79–105) → 94 bpm (68–108) → 90 bpm (68–101)	NDR	Pre → post intervention → 3-month post intervention: 52.6 ms (26.9–85.8) → 58.0 ms (25.8–102.7) → 54.2 ms (26.0–128.7)	Pre → post intervention → 3-month post intervention: 38.2 ms (16.9–71.6) → 40.6 ms (17.7–93.1) → 47.4 ms (18.1–82.0)	NDR
Kim et al., 2017 [56]	97 (58–133) (units not reported)	NDR	47.5 ms (21.3–57.7)	42.4 ms (12.2–61.4)	NDR
Amichai et al., 2019 [12]	Rest supine → rest sitting → paced breathing sitting: CP: 90.5 ± 12.3 bpm → 96.2 ± 14.1 bpm → 95.2 ± 13.5 bpm CG: 81.0 ± 11.9 bpm → 85.2 ± 14.8 bpm → 82.8 ± 12.3 bpm	NDR	Rest supine → rest sitting → paced breathing sitting: CP: 61.9 ± 25.6 ms → 50.7 ± 22.1 ms → 61.3 ± 32.6 ms CG: 72.7 ± 28.1 ms → 78.9 ± 35.0 ms → 87.1 ± 32.1 ms	Rest supine → rest sitting → paced breathing sitting: CP: 43.7 ± 19.9 ms → 26.1 ± 12.8 ms → 41.2 ± 22.1 ms CG: 60.3 ± 23.1 ms → 40.4 ± 17.1 ms → 55.4 ± 20.3 ms	NDR
Katz-Leurer et al., 2019 [14]	NDR	CP: 612 ms (540–676)<br>GMFCS I: 655 ms (610–723)<br>GMFCS II: 621 ms (570–667)<br>GMFCS III: 632 ms (543–718)<br>GMFCS IV: 536 ms (480–567)<br>GMFCS V: 574 ms (515–630)<br>CG: 729 ms (645–796)	CP: 53 ms (40–69)<br>GMFCS I: 57 ms (43–73)<br>GMFCS II: 48 ms (39–63)<br>GMFCS III: 59 ms (35–77)<br>GMFCS IV: 44 ms (40–59)<br>GMFCS V: 49 ms (38–61)<br>CG: 79 ms (68–103)	CP: 33 ms (22–46)<br>GMFCS I: 39 ms (32–51)<br>GMFCS II: 32 ms (25–35)<br>GMFCS III: 34 ms (13–48)<br>GMFCS IV: 22 ms (12–34)<br>GMFCS V: 29 ms (21–52)<br>CG: 52 ms (37–79)	NDR
Landis et al., 2019 [54]	3 gaming conditions for collection objects: I) hand icons only; II) feet icons only; III) hand and feet icons: Rest → Warmup (40-60% mean HR)	3 gaming conditions for collection objects: I) hand icons only; II) feet icons only; III) hand and feet icons: Rest →	3 gaming conditions for collection objects: 1) hand icons only; 2) feet icons only; 3) hand and feet icons: Rest → Warmup (40-60% mean	3 gaming conditions for collection objects: 1) hand icons only; 2) feet icons only; 3) hand and feet icons: Rest → Warmup	3 gaming conditions for collection objects: 1) hand icons only; 2) feet icons only; 3) hand and feet icons: Rest

	→ Conditioning 1-5 min and 6-10 min (60-80% mean HR) → Cooldown (40-60% mean HR) → Recovery: game I: 100.6 ± 13.2 bpm → 97.7 ± 11.0 bpm → 107.3 ± 12.1 bpm → 111.9 ± 12.6 bpm → 102.9 ± 11.6 bpm → 92.7 ± 10.1 bpm; game II: 97.6 ± 12.4 bpm → 95.9 ± 10.7 bpm → 103.3 ± 11.8 bpm → 106.8 ± 13.6 bpm → 99.6 ± 11.5 bpm → 92.3 ± 8.2 bpm; game III: 95.8 ± 10.7 bpm → 93.8 ± 7.7 bpm → 100.4 ± 8.5 bpm → 103.6 ± 8.8 bpm → 98.3 ± 9.7 bpm → 92.6 ± 12.8 bpm	Warmup (40-60% mean HR) → Conditioning 1-5 min and 6-10 min (60-80% mean HR) → Cooldown (40-60% mean HR) → Recovery: game I: 607.4 ± 86.5 s → 621.7 ± 75.7 s → 566.6 ± 74.5 s → 543.7 ± 72.3 s → 591.0 ± 76.2 s → 654.9 ± 81.4 s; game II: 625.1 ± 84.2 s → 633.0 ± 74.6 s → 588.7 ± 76.1 s → 571.8 ± 86.9 s → 609.6 ± 72.1 s → 654.9 ± 57.4 s; game III: 634.1 ± 70.3 s → 643.5 ± 49.1 s → 601.4 ± 50.5 s → 582.6 ± 47.7 s → 615.5 ± 57.2 s → 657.8 ± 80.7 s	HR) → Conditioning 1-5 min and 6-10 min (60-80% mean HR) → Cooldown (40-60% mean HR) → Recovery: game I: 47.4 ± 21.9 ms → 44.9 ± 19.0 ms → 37.9 ± 14.6 ms → 32.4 ± 17.4 ms → 41.9 ± 18.8 ms → 62.7 ± 24.9 ms; game II: 54.7 ± 28.9 ms → 51.6 ± 22.1 ms → 49.7 ± 20.9 ms → 48.9 ± 42.5 ms → 44.1 ± 17.7 ms → 67.8 ± 35.3 ms; game III: 59.6 ± 30.3 ms → 55.8 ± 20.5 ms → 57.2 ± 42.8 ms → 50.1 ± 33.2 ms → 50.6 ± 28.0 ms → 73.9 ± 26.3 ms	(40-60% mean HR) → Conditioning 1-5 min and 6-10 min (60-80% mean HR) → Cooldown (40-60% mean HR) → Recovery: game I: 34.9 ± 24.7 ms → 33.8 ± 23.8 ms → 28.3 ± 23.2 ms → 26.9 ± 23.5 ms → 34.4 ± 30.4 ms → 41.9 ± 26.7 ms; game II: 45.7 ± 38.8 ms → 47.6 ± 33.8 ms → 45.9 ± 33.8 ms → 53.6 ± 73.7 ms → 36.4 ± 23.8 ms → 42.9 ± 23.6 ms; game III: 49.6 ± 42.3 ms → 47.2 ± 30.0 ms → 58.6 ± 70.2 ms → 48.8 ± 46.8 ms → 44.5 ± 42.1 ms → 44.2 ± 26.7 ms	→ Warmup (40-60% mean HR) → Conditioning 1-5 min and 6-10 min (60-80% mean HR) → Cooldown (40-60% mean HR) → Recovery: game I: 10.2 ± 15.8 % → 10.2 ± 17.0 % → 7.5 ± 16.2 % → 5.1 ± 11.8 % → 8.7 ± 16.1 % → 13.3 ± 17.9 %; game II: 12.5 ± 17.9 % → 12.1 ± 18.5 % → 9.4 ± 18.3 % → 9.5 ± 20.4 % → 9.7 ± 17.3 % → 13.7 ± 14.1 %; game III: 12.6 ± 14.6 % → 12.7 ± 13.7 % → 8.8 ± 12.0 % → 6.5 ± 8.8 % → 8.9 ± 12.4 % → 16.6 ± 16.7 %

CP – cerebral palsy; CG – control group; GMFCS - Gross Motor Function Classification System; GMFM - Gross Motor Function Measure; SDNN – standard deviation of NN intervals; RMSSD – root mean square successive difference; pNN50 – percentage of adjacent NN intervals that differ from each other by more than 50 ms; bpm – beats per minute; ms – milliseconds; NDR – no data reported.

Table S2. Results for frequency domain and nonlinear HRV parameters.

First author and year of publication	LF	HF	TP	LF/HF	Nonlinear parameters
Park et al. 2002 [2]	Supine → 70º head-up tilt: Absolute [ms²] CP: 1739.8 ± 1241.7 → 1901.3 ± 1371.9 CG: 1015.4 ± 700.4 → 1358.0 ± 1175.6 Normalized [nu] CP: 51.1 ± 14.8 → 51.3 ± 17.5 CG: 41.7 ± 13.5 → 52.8 ± 15.7	Supine → 70º head-up tilt: Absolute [ms²] CP: 416.7 ± 595.0 → 1739.8 ± 1241.7 CG: 501.3 ± 472.0 → 1015.4 ± 700.4 Normalized [nu] CP: 10.4 ± 8.7 → 15.8 ± 10.2 CG: 17.0 ± 11.4 → 13.3 ± 8.3	Supine → 70º head-up tilt: Absolute [ms²] CP: 2148.8 ± 1691.1 → 2554.8 ± 1872.6 CG: 1953.3 ± 1822.7 → 1913.8 ± 1668.8	Supine → 70º head-up tilt: CP: 6.22 ± 3.69 → 5.03 ± 3.99 CG: 2.97 ± 1.98 → 5.49 ± 3.14	NDR
Yang et al. 2002 [3]	Supine → head-up tilt: Normalized [nu] CP: 52.5 ± 15.4 → 57.1 ± 10.3 CG: 43.9 ± 16.4 → 53.3 ± 14.9	Supine → head-up tilt: Normalized [nu] CP: 25.9 ± 9.9 → 25.5 ± 7.6 CG: 30.5 ± 10.9 → 26.4 ± 10.2	NDR	Supine → head-up tilt: CP: 2.61 ± 1.92 → 2.67 ± 1.72 CG: 1.87 ± 1.67 → 2.77 ± 2.56	NDR
Ferreira et al. 2011 [7]	CP: 29.1 ± 11.1 ms² CG: 24.8 ± 7.0 ms²	CP: 24.1 ± 9.8 ms² CG: 18.8 ± 6.4 ms²	CP: 86.4 ± 29.7 ms² CG: 73.5 ± 19.8 ms²	CP: 1.24 ± 0.22 CG: 1.39 ± 0.33	NDR
Zamunér et al. 2011 [5]	Supine → standing Normalized [nu] CP: 87.1 ± 20.5 → 87.7 ± 9.5 CG: 60.8 ± 17.9 → 72.3 ± 14.2	Supine → standing Normalized [nu] CP: 15.9 ± 20.5 → 12.2 ± 9.5 CG: 39.3 ± 17.9 → 27.7 ± 14.2	NDR	Supine → standing CP: 14.00 ± 11.94 → 13.95 ± 12.35 CG: 2.45 ± 2.42 → 4.45 ± 5.37	NDR
Kholod et al., 2013 [8]	NDR	NDR	NDR	NDR	NDR
Israeli-Mendlovic et al., 2014 [9]	NDR	Rest → GMFM → activity → standing: Normalized [nu] CP all: 0.36 ± 0.11 → 0.30 ± 0.13 → 0.24 ± 0.12 → 0.27 ± 0.08 GMFCS IV: 0.35 ± 0.12 → 0.26 ± 0.09 → 0.22 ± 0.14 → 0.24 ± 0.05 GMFCS V: 0.37 ± 0.09 → 0.36 ± 0.11 → 0.27 ± 0.06 → 0.30 ± 0.11	NDR	Rest → GMFM → activity → standing: CP all: 2.0 ± 1.2 → 3.0 ± 1.9 → 4.8 ± 4.4 → 3.1 ± 1.4 GMFCS IV: 2.2 ± 1.5 → 3.5 ± 1.8 → 6.0 ± 5.3 → 3.3 ± 1.0 GMFCS V: 1.8 ± 0.6 → 2.2 ± 1.3 → 2.8 ± 1.6 → 2.9 ± 1.9	NDR

Amichai et al., 2017 [11]	NDR	NDR	NDR	2.1 ± 1.4	SD1: 37.1 ± 26.2 ms<br>SD2: 80.6 ± 40.9 ms
Cohen-Holzer et al., 2017 [13]	NDR	NDR	NDR	NDR	NDR
Kim et al., 2017 [56]	Absolute [ms²]<br>296.3 (93.2–964.4)<br>Normalized [nu]<br>48.8 (29.9–64.8)	Absolute [ms²]<br>271.4 (94.4–1,062.6)<br>Normalized [nu]<br>51.2 (35.2–70.1)	Absolute [ms²]<br>1581.0 (314.9–3,167.6)	1.0 (0.4–1.8)	ApEn: 1.1 (0.7–1.3)
Amichai et al., 2019 [12]	NDR	NDR	NDR	Rest supine:<br>CP: 2.31 (0.61–11.16)<br>CG: 1.39 (0.34–5.26)	NDR
Katz-Leurer et al., 2019 [14]	NDR	NDR	NDR	CP: 1.9 (1.5–2.9)<br>GMFCS I: 1.8 (1.4–2.6)<br>GMFCS II: 1.6 (1.3–2.6)<br>GMFCS III: 1.8 (1.1–3.8)<br>GMFCS IV: 3.4 (2.0–4.9)<br>GMFCS V: 1.8 (1.3–2.4)<br>CG: 1.5 (1.0–2.1)	NDR
Landis et al., 2019 [54]	3 gaming conditions for collection objects: 1) hand icons only; 2) feet icons only; 3) hand and feet icons:<br>Units not reported<br>Rest → Warmup (40-60% mean HR) → Conditioning 1-5 min and 6-10 min (60-80% mean HR) → Cool down (40-60% mean HR) → Recovery: game I: 0.120 ± 0.002 → 0.121 ± 0.003 → 0.119 ± 0.002 → 0.118 ± 0.002 → 0.120 ± 0.002 → 0.121 ± 0.001; game II: 0.120 ± 0.002 → 0.121 ± 0.002 → 0.119 ± 0.002 → 0.119 ± 0.001 → 0.120 ± 0.002 → 0.121 ± 0.001;	3 gaming conditions for collection objects: 1) hand icons only; 2) feet icons only; 3) hand and feet icons:<br>Units not reported<br>Rest → Warmup (40-60% mean HR) → Conditioning 1-5 min and 6-10 min (60-80% mean HR) → Cool down (40-60% mean HR) → Recovery: game I: 0.169 ± 0.026 → 0.174 ± 0.022 → 0.155 ± 0.024 → 0.146 ± 0.025 → 0.164 ± 0.023 → 0.185 ± 0.019; game II: 0.175 ± 0.025 → 0.178 ± 0.022 → 0.163 ± 0.023 → 0.155 ± 0.026 → 0.171 ± 0.022 → 0.186 ± 0.016;	NDR	3 gaming conditions for collection objects: 1) hand icons only; 2) feet icons only; 3) hand and feet icons: Rest → Warmup (40-60% mean HR) → Conditioning 1-5 min and 6-10 min (60-80% mean HR) → Cool down (40-60% mean HR) → Recovery: game I (ECG): 1.278 ± 0.058 → 1.291 ± 0.032 → 1.248 ± 0.064 → 1.238 ± 0.080 → 1.296 ± 0.060 → 1.302 ± 0.031; game II (ECG): 1.218 ± 0.132 → 1.258 ± 0.074 → 1.155 ± 0.166 → 1.153 ± 0.170 → 1.192 ± 0.155	NDR

	game III: 0.120 ± 0.002 → 0.121 ± 0.001 → 0.119 ± 0.001 → 0.119 ± 0.002 → 0.120 ± 0.001 → 0.121 ± 0.002	game III: 0.179 ± 0.022 → 0.182 ± 0.015 → 0.169 ± 0.017 → 0.163 ± 0.018 → 0.173 ± 0.020 → 0.186 ± 0.027		→ 1.267 ± 0.077; game III (ECG): 1.230 ± 0.141 → 1.248 ± 0.074 → 1.194 ± 0.185 → 1.174 ± 0.220 → 1.196 ± 0.153 → 1.293 ± 0.053; game I (RR): 0.727 ± 0.102 → 0.703 ± 0.075 → 0.782 ± 0.101 → 0.824 ± 0.111 → 0.739 ± 0.087 → 0.660 ± 0.057; game II (RR): 0.702 ± 0.094 → 0.688 ± 0.074 → 0.746 ± 0.091 → 0.780 ± 0.114 → 0.711 ± 0.087 → 0.657 ± 0.053; game III (RR): 0.684 ± 0.084 → 0.670 ± 0.055 → 0.715 ± 0.074 → 0.737 ± 0.077 → 0.701 ± 0.081 → 0.666 ± 0.115	

CP – cerebral palsy; CG – control group; GMFCS - Gross Motor Function Classification System; GMFM - Gross Motor Function Measure; LF – low frequency; HF – high frequency; TP – total power; SD1 – standard deviation of the distance of each point from the y = x axis, specifies the ellipse's width; SD2 – standard deviation of each point from the y = x + average R–R interval, specifies the ellipse's length; ApEn – approximate entropy; ms – milliseconds, nu – normalized units; NDR – no data reported.

References in the Tables S1 and S2 cited in the Supplementary Materials are from the reference list of the main text:

[2]. Park, E.S.; Park, C.I.; Cho, S.-R.; Lee, J.-W.; Kim, E.J. Assessment of Autonomic Nervous System with Analysis of Heart Rate Variability in Children with Spastic Cerebral Palsy. *Yonsei Med J*. **2002**, *43*, 65–72. doi:10.3349/ymj.2002.43.1.65.

[3]. Yang, T.F.; Chan, R.C.; Kao, C.L.; Chiu, J.W.; Liu, T.J.; Kao, N.T.; Kuo, T.B.J. Power Spectrum Analysis of Heart Rate Variability for Cerebral Palsy Patients. *Am. J. Phys. Med. Rehabil*. **2002**, *81*, 350–354. doi:10.1097/00002060-200205000-00005.

[5]. Zamunér, A.R.; Cunha, A.B.; Da Silva, E.; Negri, A.P.; Tudella, E.; Moreno, M.A. The influence of motor impairment on autonomic heart rate modulation among children with cerebral palsy. *Res. Dev. Disabil*. **2011**, *32*, 217–221. doi:10.1016/j.ridd.2010.09.020.

[7]. Ferreira, M.; Pastore, C.A.; Imada, R.; Guare, R.O.; Leite, M.; Poyares, D.; Santos, M.T.B.R. Autonomic nervous system in individuals with cerebral palsy: A controlled study. *J. Oral Pathol. Med*. **2011**, *40*, 576–581. doi:10.1111/j.1600-0714.2011.01008.x.

[8]. Kholod, H.; Jamil, A.; Katz-Leurer, M. The associations between motor ability, walking activity and heart rate and heart rate variability parameters among children with cerebral palsy and typically developed controls. *Neurorehabilitation* **2013**, *33*, 113–119. doi:10.3233/nre-130934.

[9]. Israeli-Mendlovic, H.; Mendlovic, J.; Katz-Leurer, M. Heart rate and heart rate variability parameters at rest, during activity and passive standing among children with cerebral palsy GMFCS IV–V. *Dev. Neurorehabilit*. **2014**, *17*, 398–402. doi:10.3109/17518423.2014.895439.

[11]. Amichai, T.; Eylon, S.; Dor-Haim, H.; Berger, I.; Katz-Leurer, M. Cardiac Autonomic System Response to Submaximal Test in Children with Cerebral Palsy. *Pediatr. Phys. Ther.* **2017**, *29*, 125–128. doi:10.1097/pep.0000000000000368.

[12]. Amichai, T.; Eylon, S.; Berger, I.; Katz-Leurer, M. The impact of breathing rate on the cardiac autonomic dynamics among children with cerebral palsy compared to typically developed controls. *Dev. Neurorehabilit*. **2018**, *22*, 98–103. doi:10.1080/17518423.2018.1434700.

[13]. Cohen-Holzer, M.; Sorek, G.; Schweizer, M.; Katz-Leurer, M. The influence of a constraint and bimanual training program using a variety of modalities on endurance and on the cardiac autonomic regulation system of children with unilateral cerebral palsy: A self-control clinical trial. *Neurorehabilitation* **2017**, *41*, 119–126. doi:10.3233/nre-171463.

[14]. Katz-Leurer, M.; Amichai, T. Heart rate variability in children with cerebral palsy. *Dev. Med. Child Neurol*. **2019**, *61*, 730–731. doi:10.1111/dmcn.14095.

[54]. Landis, C.; O'Neil, M.E.; Finnegan, A.; Shewokis, P.A. Calculating Heart Rate Variability from ECG Data from Youth with Cerebral Palsy During Active Video Game Sessions. *J. Vis. Exp.* **2019**, e59230. doi:10.3791/59230.

[56]. Kim, S.W.; Jeon, H.R.; Kim, J.; Kim, Y. Heart Rate Variability Among Children With Acquired Brain Injury. *Ann. Phys. Rehabil. Med*. **2017**, *41*, 951–960. doi:10.5535/arm.2017.41.6.951.
